# Risk subtyping and prognostic assessment of prostate cancer based on consensus genes

**DOI:** 10.1038/s42003-022-03164-8

**Published:** 2022-03-15

**Authors:** Jialin Meng, Yu Guan, Bijun Wang, Lei Chen, Junyi Chen, Meng Zhang, Chaozhao Liang

**Affiliations:** 1 Department of Urology, The First Affiliated Hospital of Anhui Medical University, 218th Jixi Road, 230022 Hefei, Anhui People’s Republic of China; 2 Institute of Urology, Anhui Medical University, 218th Jixi Road, 230022 Hefei, Anhui People’s Republic of China; 3 Anhui Province Key Laboratory of Genitourinary Diseases, Anhui Medical University, 218th Jixi Road, 230022 Hefei, Anhui People’s Republic of China; 4 Urology Institute of Shenzhen University, The Third Affiliated Hospital of Shenzhen University, Shenzhen University, 518000 Shenzhen, Guangdong People’s Republic of China

**Keywords:** Urological cancer, Prostate

## Abstract

Prostate cancer (PCa) is the most frequent malignancy in male urogenital system around worldwide. We performed molecular subtyping and prognostic assessment based on consensus genes in patients with PCa. Five cohorts containing 1,046 PCa patients with RNA expression profiles and recorded clinical follow-up information were included. Univariate, multivariate Cox regression analysis and least absolute shrinkage and selection operator (LASSO) Cox regression were used to select prognostic genes and establish the signature. Immunohistochemistry staining, cell proliferation, migration and invasion assays were used to assess the biological functions of key genes. Thirty-nine intersecting consensus prognostic genes from five independent cohorts were identified. Subsequently, an eleven-consensus-gene classifier was established. In addition, multivariate Cox regression analyses showed that the classifier served as an independent indicator of recurrence-free survival in three of the five cohorts. Combined receiver operating characteristic (ROC) analysis achieved synthesized effects by combining the classifier with clinicopathological features in four of five cohorts. SRD5A2 inhibits cell proliferation, while ITGA11 promotes cell migration and invasion, possibly through the PI3K/AKT signaling pathway. To conclude, we established and validated an eleven-consensus-gene classifier, which may add prognostic value to the currently available staging system.

## Abstract (web summary)

By analysis of gene expression profiles of prostate cancer patients from multiple platforms, an eleven-consensus-gene classifier is constructed to provide a robust tool for the prediction of recurrence-free survival.

## Introduction

Prostate cancer (PCa) is the most frequent malignancy in male urogenital system around worldwide^1,2^. Although the majority of localized PCa patients can be cured by surgery and/or radiation therapy, some PCa patients still face the severe scenario of progressing to castration-resistant PCa (CRPC)^3,4^. In contrast, some patients have indolent tumors, which rarely progress to an advanced stage or influence their quality of life. Thus, it is crucial to recognize the potential risk of recurrence for patients before therapy.

The Gleason score is a widely used feature to reflect the degree of malignancy for PCa^5–8^. Along with the published studies, patients with Gleason score ≤6 rarely suffer threat of death, while those patients with Gleason score >8 frequently confront the fact of tumor progression^9^. Although histological examination easily discriminates tumors with Gleason score <6 or >8, it is still difficult to verify patients with an intermediate stage (Gleason score 3 + 4 or 4 + 3)^10^. Commonly, PCa patients with Gleason score of 3 + 4 are less aggressive than those with Gleason score of 4 + 3. However, sampling error, bias among separate pathologists, and the subjectivity of assessments are clear confounding factors for misleading results^11^. Therefore, it is urgent to develop a universal molecular classifier to recognize patients with a high risk of poor prognosis.

Since the early 2000s, gene-expression profiles have been applied to classify early-stage tumor patients into distinct subtypes based on molecular markers, and these subtypes of patients were correlated with diverse outcomes or clinicopathological features. Furthermore, studies also indicated that the gene-expression panel test could guide tailored treatment decisions for doctors, promoting the development of personalized medicine^12^. Recently, increasing evidence has shown that gene-expression-based classifiers may be useful for disease classification independent of the available prognostic factors, serving as clinical implementations. Although the gene-expression classifier could be constructed and validated in the publicly released dataset or even a single-center study, there remains a gap invalidating the identified classifier in large cohorts.

We aimed to assess the usage of consensus recurrence-free survival (RFS)-associated genes derived from five independent cohorts to generate molecular subtyping with different clinical outcomes for PCa patients.

## Results

### Construction of the eleven-consensus-gene classifier

**Fig. 1:**
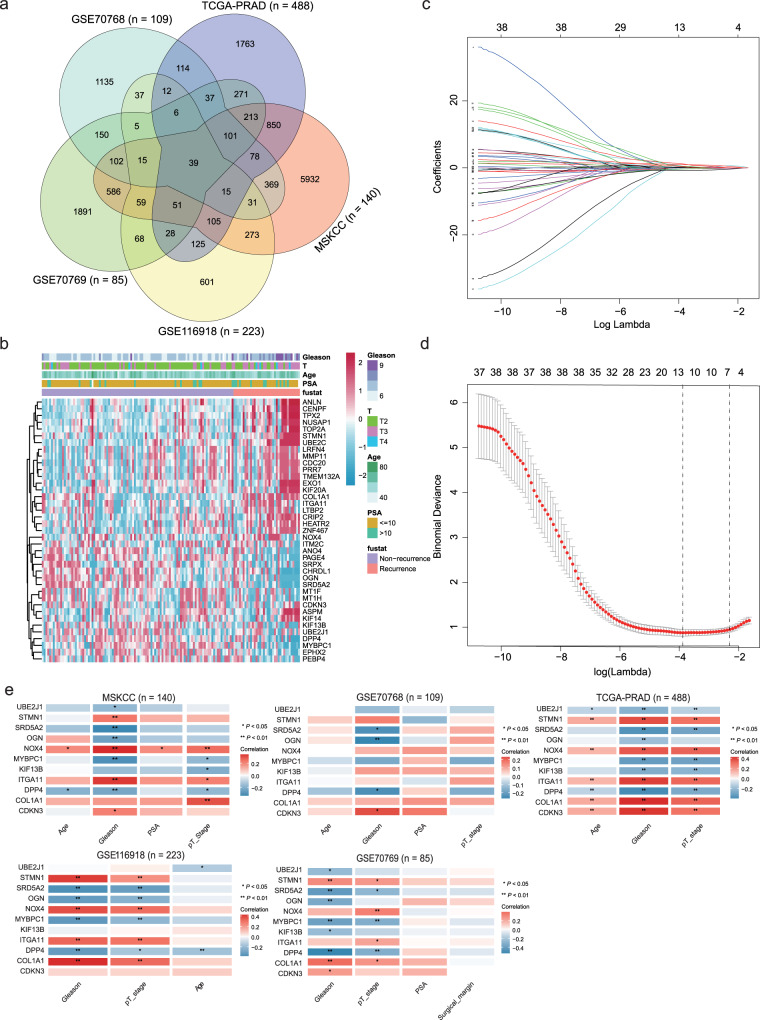
Recognizing of the consensus recurrence-free survival-related genes and establishing the classifier. **a** Venn diagram showing the 39 overlapping recurrence-free survival (RFS)-associated genes with a *P-*value < 0.05 in the five independent datasets. **b** Heatmap showing the expression landscape of the 39 RFS-related genes in the MSKCC dataset; blanks/gaps indicate missing values. **c** LASSO analysis revealed the coefficients in the model at varying levels of penalization plotted against the log (lambda) sequence. **d** Partial likelihood deviance was plotted versus log (lambda). **e** Correlation analyses between the expression of the eleven consensus genes and clinicopathological features in the MSKCC, GSE70768, TCGA-PRAD, GSE116918, and GSE70769 cohorts. *P < 0.05, **P < 0.01.

In the current study, we enrolled a total of 1046 PCa patients from the MSKCC (*n* = 140), TCGA-PRAD (*n* = 488), GSE116918 (*n* = 223), GSE70769 (*n* = 85), and GSE70768 (*n* = 109) datasets. The clinicopathological features of all the PCa patients are listed in Table 1. We overlapped the significant genes (*P* < 0.05) generated by univariate Cox regression analyses in five independent cohorts and found that there were 39-consensus candidates and significantly associated with the RFS of PCa patients in all five datasets (Fig. 1a, and Supplementary Table 1). The expression landscape of these 39 genes in the MSKCC cohort is shown in Fig. 1b. To obtain a more stable and significant consensus-gene-based classifier, we employed LASSO Cox analysis based on the MSKCC dataset. Finally, 11 RFS-related consensus genes were selected, including *MYBPC1*, *DPP4*, *UBE2J1*, *KIF13B*, *SRD5A2*, *OGN*, *NOX4*, *ITGA11*, *COL1A1*, *STMN1*, and *CDKN3* (Fig. 1c, d). Then, the risk score of each patient was calculated by the prognostic model: 0.541407161 * *CDKN3* + 0.986301077 * *COL1A1* + 0.055793216 * *DPP4* + 1.204285151 * *ITGA11* − 0.329370122 * *KIF13B* − 0.353092775 * *MYBPC1* + 0.228495902 * *NOX4* − 0.374366498 * *OGN* − 0.711376668 * *SRD5A2* + 0.492742742 * *STMN1* + 0.417671671 * *UBE2J1*.

**Table 1:** Summary of the clinicopathological parameters of five independent prostate cancer datasets.

Items	MSKCC (*n* = 140)	TCGA-PRAD (*n* = 488)	GSE70768 (*n* = 109)	GSE70769 (*n* = 85)	GSE116918 (*n* = 223)
Age					
<60	87	219	42	—	26
≥60	53	269	67	—	197
Pathology T grade					
T1 + T2	86	187	34	46	127
T3 + T4	54	301	75	39	96
Gleason^a^					
5	—	—	—	2	—
6	41	45	17	17	39
7	76	246	83	53	88
8	11	63	8	5	47
9	10	137	1	7	49
10	—	3	—	1	—
ISUP group^b^					
1	41	42	17	19	—
2	53	143	62	34	—
3	23	100	21	19	—
4	11	63	8	5	—
5	10	140	1	8	—
EAU group^c^					
Low	38	42	11	14	—
Middle	73	226	70	50	—
High	27	220	27	21	—
PSA^d^					
>10 ng/ml	24	15	26	26	—
≤10 ng/ml	114	416	82	59	—
PCa type					
Primary	131	488	109	85	223
Metastasis	9	0	0	0	0
Recurrence rate	25.71%	19.06%	17.43%	48.24%	22.87%

^a, b, c^Gleason score information is missing in two patients from MSKCC cohort.

^d^PSA data are missing in two patients from MSKCC cohort, in 57 patients from TCGA-PRAD cohort, in one patient from GSE70768 cohort.

The median risk score was set as the cutoff value in each cohort, and these patients with lower-risk scores were assigned to the low-risk subgroup, while others were classified into the high-risk subgroup (Supplementary Data 1). Furthermore, we correlated the expression of the 11 genes with clinicopathological features, and the results indicated that *UBE2J1*, *SRD5A2*, *OGN*, *MYBPC1*, *KIF13B*, and *DPP4* were negatively correlated with Gleason score, PSA level, and pathological tumor stage, while opposite results were obtained for the *STMN1*, *NOX4*, *ITGA11*, *COL1A1*, and *CDKN3* genes (Fig. 1e).

### Prognostic assessment of the eleven-consensus-gene classifier in five cohorts

**Fig. 2:**
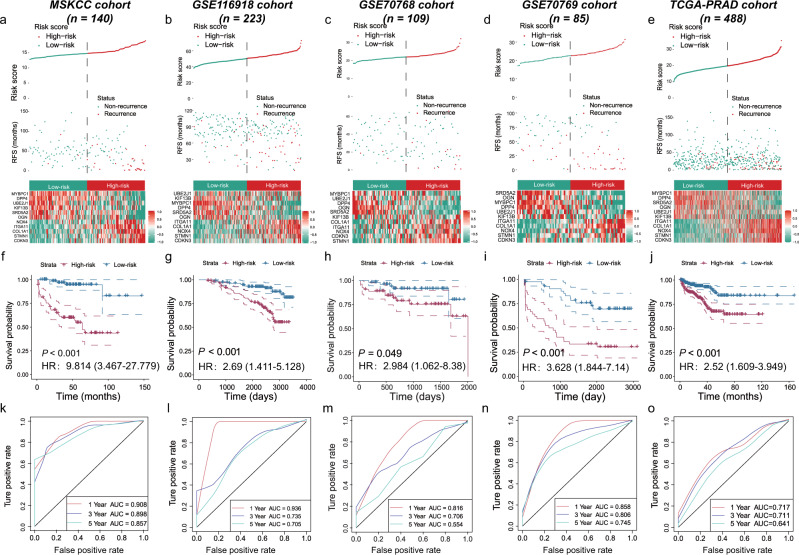
The prognostic value of the eleven-consensus-gene classifier. **a**–**e** The plots display the risk classification (upper), corresponding to the distribution of real recurrent samples (middle), and the heatmap displays the expression of the 11 candidates in distinct risk subgroups (lower) in five cohorts. **f**–**j** Kaplan–Meier plots showed the distinguishing value of favorable and poor recurrence-free survival in five cohorts. **k**–**o** ROC curves showed the predictive accuracy of the eleven-consensus-gene-based classifier for RFS prognosis in five cohorts.

Referring to the subgroups, in the training MSKCC cohort, 70 patients were divided into the high-risk group, and the other 70 patients belonged to the low-risk group (Fig. 2a). Patients in the high-risk subgroup showed an unfavorable prognosis (log-rank *P* < 0.001, Fig. 2f), with AUC values of 0.908 at 1 year, 0.898 at 3 years, and 0.857 at 5 years (Fig. 2k). The predicting classifier also applied well in four external datasets. The K–M curves showed similar RFS outcomes in the GSE116918 (log-rank, *P* < 0.001), GSE70768 (log-rank, *P* = 0.049), GSE70769 (log-rank, *P* < 0.001), and TCGA-PRAD cohorts (log-rank, *P* < 0.001) (Fig. 2b–e, g–j). The classifier showed moderate predictive accuracy in all four validation cohorts (AUC values of 0.936, 0.735, and 0.705 at 1, 3, and 5 years in the GSE116918 cohort; AUC values of 0.816, 0.706, and 0.554 at 1, 3, and 5 years in the GSE70768 cohort; AUC values of 0.858, 0.806, and 0.745 at 1, 3, and 5 years in the GSE70769 cohort; AUC values of 0.717, 0.711, and 0.641 at 1, 3, and 5 years in the TCGA-PRAD cohort, Fig. 2l–o). We also validated the prognostic value of the eleven-consensus-gene classifier in the external GSE46602 cohort, and we revealed that patients with higher-risk score had a poor prognosis (*P* = 0.033, HR = 3.55, 95% CI = 1.108–11.385), with a prognostic AUC value of 0.760 (Supplementary Fig. 1).

To further investigate the clinical application value of the eleven-consensus-gene classifier, we performed the K–M analyses in different clinicopathological subgroups. The signature precisely subclassified the high- and low-risk groups of PCa patients into different subgroups with an adequate number of samples but failed in some conditions, potentially attributing to the small sample size (Supplementary Fig. 2). For example, in the MSKCC cohort, which comprised 140 PCa cases, the results suggested that the classifier significantly discriminated the high- and low-risk subgroups in separate age (≥60 vs. <60 years old), PSA level (>10 vs. ≤10 ng/dl), pathological tumor stage (T3 + T4 vs. T1 + T2), and Gleason score (>7 vs. ≤7) subgroups (all, log-rank *P* < 0.05). For the GSE70769 cohort, which comprised 85 PCa cases, we also revealed that the classifier significantly discriminated the high- and low-risk subgroups of the different PSA (>10 vs. ≤10), tumor stage (T3 + T4 vs. T1 + T2) subgroups (log-rank *P* < 0.05), Gleason score (>7 vs. ≤7) subgroups, and surgical margin (negative vs. positive) (all, log-rank *P* < 0.05). Further clinical trial is warranted to verify our findings.

Notably, the clinical outcomes of PCa patients with Gleason score 3 + 4 and 4 + 3 were different. It is necessary to distinguish these two subgroups with not only pathological results. We investigated the value of this classifier in distinguishing patients with Gleason score 3 + 4 and 4 + 3 subgroups in the MSKCC, TCGA-PRAD, GSE70768, and GSE70769 datasets, while GSE116918 missed the information of primary and secondary Gleason score was removed from the analysis. We observed that patients with a Gleason score of 4 + 3 had a higher-risk score than patients with a Gleason score of 3 + 4 (MSKCC, *P* = 0.092; TCGA-PRAD, *P* < 0.001; GSE70768, *P* = 0.006; GSE70769, *P* = 0.007). In addition, the risk score distinguished the 3 + 4/4 + 3 subgroups with good accuracy (MSKCC, AUC = 0.636; TCGA-PRAD, AUC = 0.718; GSE70768, AUC = 0.712; GSE70769, AUC = 0.731). As confirmed by Fisher’s extract test, we found that more patients with high risk belonged to the Gleason score 4 + 3 subgroup (MSKCC, *P* = 0.079; TCGA-PRAD, *P* < 0.001; GSE70768, *P* = 0.024; GSE70769, *P* = 0.010) (Supplementary Fig. 3).

### Analysis results of multivariate Cox regression and combined ROC

**Fig. 3:**
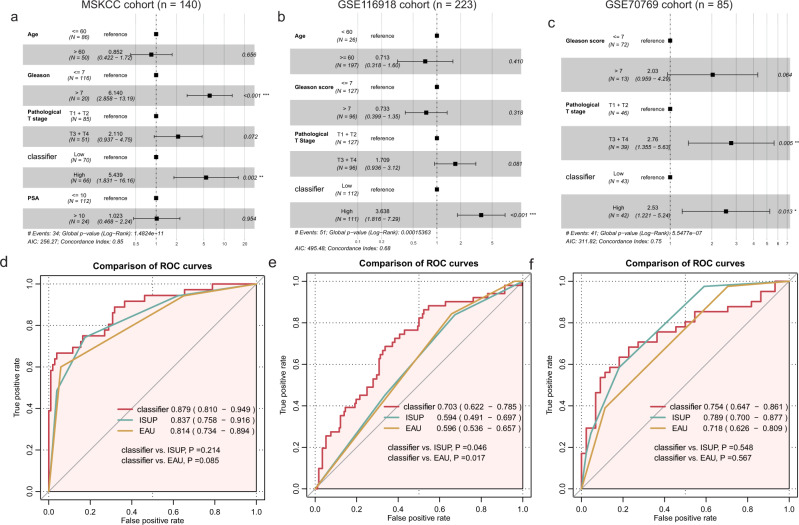
Multivariate regression and combined receiver operating characteristic curve analyses. Forest plot showing the independent prognostic value of the classifier in the MSKCC (**a**), GSE116918 (**b**), and GSE70769 (**c**) cohorts; Comparison of the prognostic value of clinicopathological features, classifier and the synthesized model by receive operating characteristic curve in the MSKCC (**d**), GSE116918 (**e**), and GSE70769 (**f**) cohorts. *P < 0.05, **P < 0.01, ***P < 0.001.

To determine the independence of the eleven-consensus-gene classifier in each cohort, we performed multivariate Cox regression analyses. Our results showed that for the cohorts whose recurrence rate >15%, the classifier served as an independent indicator for RFS (MSKCC cohort: HR = 5.44, 95% CI: 1.83–16.16, *P* = 0.002; GSE116918 cohort: HR = 3.64, 95% CI: 1.82–7.29, *P* < 0.001; GSE70769 cohort: HR = 2.53, 95% CI: 1.22–5.24, *P* = 0.013; TCGA-PRAD cohort: HR = 1.39, 95% CI: 0.76–2.52, *P* = 0.282; GSE70768 cohort: HR = 0.818, 95% CI: 0.29–2.28, *P* = 0.701; Fig. 3a–c, and Supplementary Fig. 4a, b).

We also compared the prognostic value of eleven-consensus-gene classifier, ISUP, EAU risk group by the ROC curve, it is gratifying that the eleven-consensus-gene classifier showed a comparable prognostic value to ISUP, EAU risk group in MSKCC cohort (AUC value, classifier: 0.879 vs. ISUP: 0.837 vs. EAU: 0.814; Comparison *P*-value, classifier vs. ISUP: *P* = 0.214, classifier vs. EAU: *P* = 0.085, Fig. 3d), GSE70769 cohort (AUC value, classifier: 0.754 vs. ISUP: 0.789 vs. EAU: 0.718; Comparison *P*-value, classifier vs. ISUP: *P* = 0.548, classifier vs. EAU: *P* = 0.567, Fig. 3f), TCGA-PRAD cohort (AUC value, classifier: 0.720 vs. ISUP: 0.732 vs. EAU: 0.690; Comparison *P*-value, classifier vs. ISUP: *P* = 0.684, classifier vs. EAU: *P* = 0.423, Supplementary Fig. 4c), GSE70768 cohort (AUC value, classifier: 0.721 vs. ISUP: 0.721 vs. EAU: 0.591; Comparison *P*-value, classifier vs. ISUP: *P* = 0.997, classifier vs. EAU: *P* = 0.052, Supplementary Fig. 4c), and a preferable prognostic value in GSE116918 cohort (AUC value, classifier: 0.703vs. ISUP: 0.594 vs. EAU: 0.596; Comparison *P*-value, classifier vs. ISUP: *P* = 0.046, classifier vs. EAU: *P* = 0.017, Fig. 3e). Taken together, the eleven-consensus-gene classifier showed a comparable prognostic value with ISUP and EAU risk group.

### IHC validation the protein of SRD5A2 and ITGA11

We chose *SRD5A2* and *ITGA11* for further validation, due to the high weight of these two genes in the risk score formula, 1.20428 for *ITGA11* and 0.71137 for *SRD5A2*. What’s more, several studies reported the association between *SRD5A2* polymorphism and PCa risk, while rarely study reported the function of ITGA11 in PCa, therefore, we final chose these two genes for experimental validation. To address and confirm the associations of SRD5A2 and ITGA11 protein levels with clinicopathological features, we used IHC assay on a prostate cancer tissue array, which contains tumor tissues from 42 patients. The standard definition of the SRD5A2 protein level is described in the Methods section. Then, we calculated the staining density of each tissue. Tissues with score equal to or higher than 3 were regarded as positive, while those with score less than 3 were negative.

**Fig. 4:**
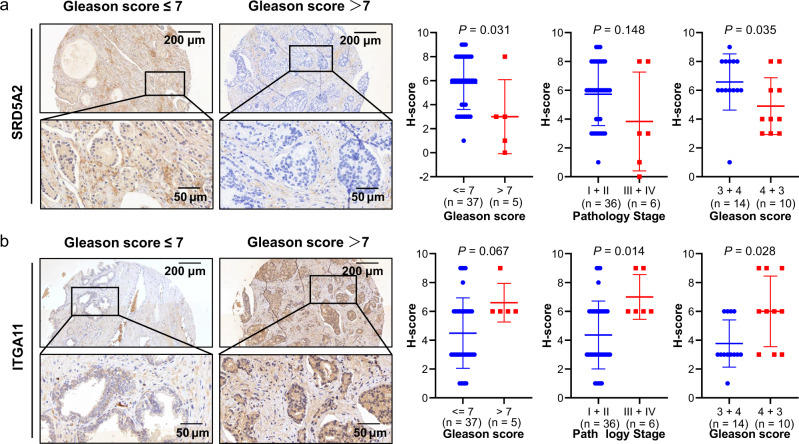
Immunohistochemistry validation of SRD5A2 and ITGA11 expression in PCa tumor tissues. **a** Representative pictures showing the different protein levels of SRD5A2 in prostate cancer patients with different Gleason scores. Quantitative comparisons of H-scores were also performed in Gleason and pathological tumor stage subgroups. **b** Representative pictures showing the different protein levels of ITGA11 in prostate cancer patients with different Gleason scores. Quantitative comparison of staining density was also performed in Gleason and pathological tumor stage subgroups. Data are presented as the mean ± SD by *t*-test, bars represent mean values, error bars represent SD. Scale bars, 50 μm, 200 μm.

We observed decreased protein expression of SRD5A2 in advanced tumor stages (Gleason ≤ 7 vs. Gleason >7, *P* = 0.031, Stage I + II vs. Stage III + IV, *P* = 0.148, Gleason 3 + 4 vs. Gleason 4 + 3, *P* = 0.035, Fig. 4a). Moreover, with the help of Fisher’s extract test, we investigated the different distributions of SRD5A2 expression (strong positive, weak positive, or negative) in different clinicopathological subgroups. We revealed a negative association of SRD5A2 and tumor progression, reflected by the Gleason score (*P* = 0.013) and pathological tumor stage (*P* = 0.047) (Table 2). Regarding ITGA11, we observed elevated protein expression in the advanced stage compared with the early stage (Gleason ≤ 7 vs. Gleason > 7, *P* = 0.067, Stage I + II vs. Stage III + IV, *P* = 0.014, Gleason 3 + 4 vs. Gleason 4 + 3, *P* = 0.028, Fig. 4b). Fisher’s extract test of the categorical variables illustrated similar results. These patients whose Gleason score was higher than 7 or who were in tumor stages III and IV showed strong positive staining of ITGA11 (Gleason score, *P* = 0.049, pathological tumor stage, *P* = 0.022, Table 3). All these results indicated that the SRD5A2 protein is negatively associated with the progression of PCa, while the ITGA11 protein is positively associated with the advanced stage.

**Table 2:** Association between SRD5A2 protein level and pathological features in tissue array.

Parameter	IHC results for SRD5A2			
	Strong positive (*n*, %)	Weak positive (*n*, %)	Negative (*n*, %)	*P-*value
Age				0.357
<60	4 (66.67%)	1 (16.67%)	1 (16.67%)	
≥60	22 (61.11%)	12 (33.33%)	2 (5.56%)	
Envelope invasion				0.304
No	24 (64.86%)	11 (29.73%)	2 (5.41%)	
Yes	2 (40.00%)	2 (40.00%)	1 (20.00%)	
Seminal vesicle invasion				0.395
No	26 (63.41%)	12 (29.27%)	3 (7.32%)	
Yes	0 (0.00%)	1 (100.00%)	0 (0.00%)	
Gleason score				0.013*
≤7	25 (67.57%)	11 (29.73%)	1 (2.70%)	
>7	1 (20.00%)	2 (40.00%)	2 (40.00%)	
Pathology stage				0.047*
I–II	24 (66.67%)	11 (30.56%)	1 (2.78%)	
III–IV	2 (33.33%)	2 (33.33%)	2 (33.33%)	

*P < 0.05.

**Table 3:** Association between ITGA11 protein level and pathological features in tissue microarray.

Parameter	IHC results for ITGA11			
	Strong positive (*n*, %)	Weak positive (*n*, %)	Negative (*n*, %)	*P-*value
Age				0.461
<60	2 (33.33%)	3 (50.00%)	1 (16.67%)	
≥60	20 (55.56%)	12 (33.33%)	4 (11.11%)	
Envelope invasion				0.667
No	19 (51.35%)	14 (37.84%)	4 (10.81%)	
Yes	3 (60.00%)	1 (20.00%)	1 (20.00%)	
Seminal vesicle invasion				1.000
No	21 (51.22%)	15 (36.59%)	5 (12.20%)	
Yes	1 (100.00%)	0 (0.00%)	0 (0.00%)	
Gleason score				0.049*
≤7	17 (45.95%)	20 (54.05%)		
>7	5 (100.00%)	0 (0.00%)		
Pathology stage				0.022*
I–II	16 (44.44%)	20 (55.56%)		
III–IV	6 (100.00%)	0 (0.00%)		

*P < 0.05.

### Knockdown of SRD5A2 and ITGA11 impacts prostate cancer cell behaviors

**Fig. 5:**
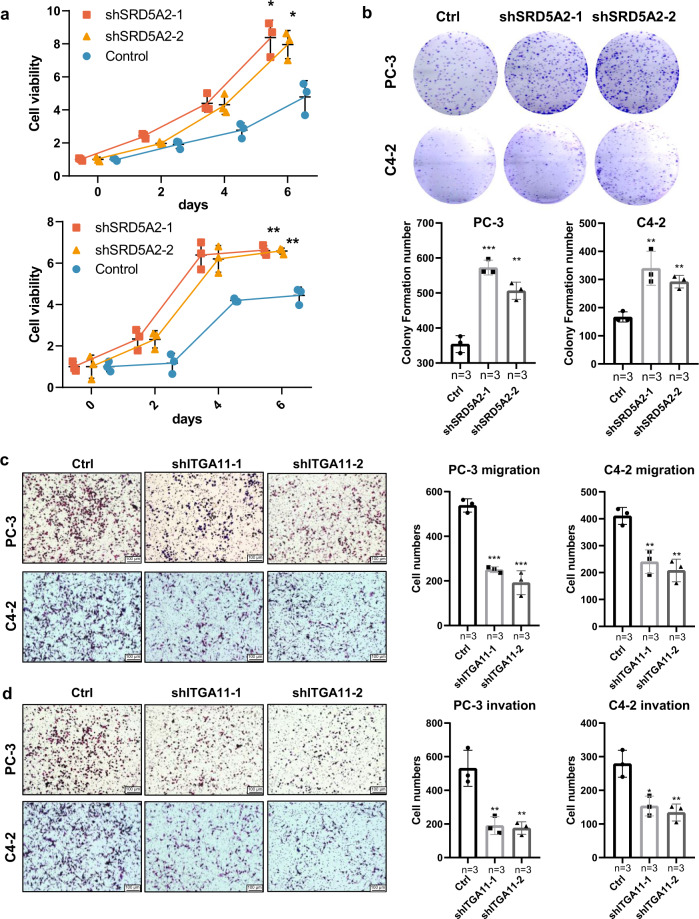
Knockdown OF SRD5A2 and ITGA11 alters cell proliferation, migration, and invasion. Comparison of cell proliferation among the control and knockdown SRD5A2 groups in PC-3 and C4-2 cell lines by MTT assay (**a**) and colony formation assay (**b**). Comparison of cell migration (**c**) and invasion (**d**) among the control and knockdown ITGA11 groups in PC-3 and C4-2 cell lines. Data are presented as the mean ± SD based on three independent experiments (*P < 0.05, **P < 0.01, ***P < 0.001 by t-test), bars represent mean values, error bars represent SD. Scale bars, 100 μm.

After knocking down the expression of SRD5A2 (*P* < 0.05, Supplementary Fig. 5), we found that cell proliferation was significantly increased, as determined by MTT assay and colony formation assays, in C4-2 and PC-3 cells (all *P* < 0.05, Fig. 5a, b). Since the functional role of SRD5A2 in regulating PCa cell migration and invasion has been investigated by Suruchi Aggarwal et al.^13^, we only focused on the proliferation effects here. In contrast, we found that silencing ITGA11 expression decreased the migration and invasion of C4-2 and PC-3 cells but not cell proliferation (all *P* < 0.05, Fig. 5c, d). These results confirm the tumor-suppressive role and oncogenic role of SRD5A2 and ITGA11, respectively.

### Exploring the underlying mechanisms of how ITGA11 regulates PCa progression

**Fig. 6:**
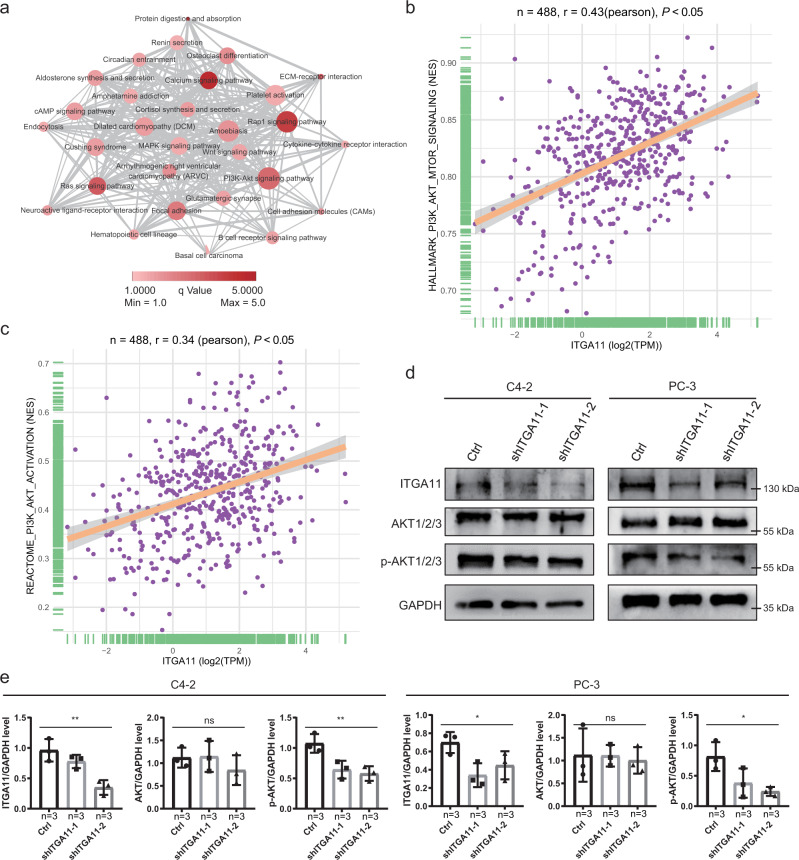
ITGA11 inhibits prostate cancer cell migration and invasion through PI3K/AKT signaling. **a** Overlapping coexpressed genes of ITGA11 in five cohorts and KEGG pathway enrichment analysis. **b** Correlation between ITGA11 expression and the activation level of PI3K/AKT signaling assessed by the HALLMARK PI3K/AKT/mTOR signaling signature. **c** Correlation between ITGA11 expression and the activation level of PI3K/AKT signaling assessed by the REACTOME PI3K/AKT activation signature. **d** Western blot analysis validated the inhibition of Ser473 p-AKT1/2/3 activation via knockdown of shITGA11 in both C4-2 and PC-3 cells. **e** Quantification of the western blotting results showing in **d**. Data are presented as the mean ± SD based on three independent experiments (ns not significant, *P < 0.05, **P < 0.01 by one-way ANOVA), bars represent mean values, error bars represent SD.

Many studies have already demonstrated the mechanisms of how the selected 11 genes influence the progression of PCa, while few studies have focused on the role of ITGA11. We conducted Pearson correlation analyses to identify the highly coexpressed genes in each cohort and overlapped these genes derived from all five cohorts. Then, KEGG analysis was used to enrich the significant signaling pathways. We found that ITGA11 might be involved in the regulation of calcium signaling, Rap1 signaling, Ras signaling, and PI3K/Akt signaling pathways (Fig. 6a). Moreover, we collected two gene sets that could reflect the activation status of PI3K/AKT signaling and calculated the NES score of each patient by ssGSEA. We observed that the elevated expression of ITGA11 was linked with the increasing NES score of the HALLMARK PI3K/AKT/mTOR signaling gene set (*P* < 0.05, r = 0.43, Fig. 6b) and the REACTOME PI3K/AKT activation gene set (*P* < 0.05, r = 0.34, Fig. 6c). In addition, we validated the function of ITGA11 in the activation of PI3K/AKT signaling in vitro. After knocking down the expression of ITGA11, we found that Ser473 phosphorylated-AKT1/2/3 was significantly decreased instead of total AKT1/2/3 (Fig. 6d, e), indicating that ITGA11 promotes the malignant phenotypes of PCa by the activating of PI3K/AKT signaling.

### Comparison between the eleven-consensus-gene classifier and proposed signatures

**Fig. 7:**
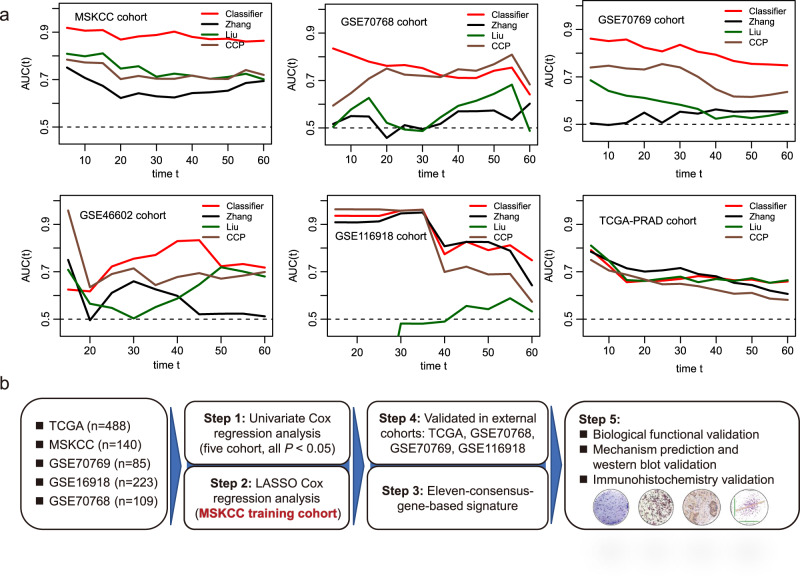
Comparing the prognostic prediction value between the eleven-consensus-gene classifier and proposed signatures. **a** Comparison of time-dependent area under the receiver operating characteristic curve value in MSKCC, GSE70768, GSE70769, GSE46602, GSE116918, and TCGA-PRAD cohorts. **b** Flow chart for the steps applied in the current study.

We calculated the risk score of the current classifier, Zhang et al.’s score, Liu et al.’s score and CCP score in the MSKCC, GSE70768, GSE70769, GSE116918, GSE46602, and TCGA-PRAD cohorts, respectively. It is gratifying that we observed that the eleven-consensus-gene classifier showed better prognostic value than the other three signatures in the MSKCC, GSE70768, GSE70769, and GSE46602 cohorts (Fig. 7a). The eleven-consensus-gene classifier showed comparable prediction efficiency with other signatures in the GSE116918 cohort and TCGA-PRAD cohort (Fig. 7a).

## Discussion

The interpatient heterogeneity in PCa is well recognized^14–16^. However, the molecular stratification of PCa based on predictive biomarkers to guide treatment selection has not yet been applied in the clinic. In our study, we analyzed five datasets derived from the GEO and TCGA databases to generate an eleven-consensus-gene classifier (Fig. 7b). We first employed univariate Cox regression analyses and identified 39 candidate genes that are closely related to the RFS of PCa patients in all five datasets. The RFS predicting classifier was established by the LASSO Cox regression analysis based MSKCC dataset. The classifier showed satisfying molecular subtyping accuracy determined by the log-rank, K–M, and ROC analyses in both the training and four external validation cohorts. Furthermore, the multivariate analyses suggested that the classifier served as an independent indicator of RFS in a set of cohorts. Notably, the combined ROC curve, which synthesized the classifier with clinicopathological features, added prognostic value to the currently available staging system. We conducted Pearson correlation analyses to determine the highly coexpressed genes in each cohort and overlapped these genes derived from all five cohorts. Then, we employed KEGG pathway analyses to reveal the underlying mechanisms of these critical candidates influencing tumor progression. For the eleven candidates, Yu et al.^17^ reported that CDKN3 downregulated the expression levels of cell-cycle- and DNA-replication-related proteins. It has also been reported that abundant miR-92a-1-5p from PCa exosomes can downregulate COL1A1 and thus promote osteoclast differentiation and inhibit osteoblast genesis^18^. Pan et al.^19^ revealed the higher level of DPP4 in malignant prostate tissue than that in benign prostate tissue, its expression correlated with PSA and tumor stage. Kamata et al.^20^ reported that zinc finger mutation of PARP7 can result in the loss of PARP7, and further impact the enhancement of AR-dependent transcription of the MYBPC1 gene. Wu et al.^21^ found that silencing of NOX4 can contribute to the decreasing of lactate production, glucose uptake, ATP production, and cell proliferation but increasing the apoptosis. In aggressive prostate cancers, the oncoprotein STMN1 is often overexpressed, Chakravarthi et al.^22^ reported that CtBP1-regulated miR-34a modulates STMN1 expression and involved in the progression of prostate cancer via the regulation of GDF15. Thus, we predicted the biological function of ITGA11 through the bioinformatic method indicated above, and the results indicated that *ITGA11* might be involved in the regulation of calcium signaling, Rap1 signaling, Ras signaling, and PI3K-Akt signaling pathway activity. Consistently, studies also reported that SRD5A2 regulates cell migration and invasion by indirectly regulating the ERK/MAPK pathway^13^. Ntais et al.^23^ also reported that the A49T and TA repeat polymorphisms of SRD5A2 can increase the PCa susceptibility to human beings, elevating the important function of SRD5A2 in PCa. We further investigated the functional role of these two candidates in regulating cancer cell fates, as well as the protein expression in clinical samples. Our results confirm the tumor-suppressive role of SRD5A2 and the oncogenic role of ITGA11 in PCa. Next, we validated the pathway enrichment results based on coexpressed genes by western blot assay. Our results suggested that silencing ITGA11 suppresses the activity of AKT signaling, indicating that ITGA11 promotes PCa cell progression potentially through activating AKT signaling. Overall, we successfully established a solid prognosis prediction system.

In recent days, several prognostic classifiers have been developed to predict the outcome of PCa patients based on clinical features, gene genetics, or epigenetics. Zhao et al.^24^ reported that PD-L2 is a prognostic biomarker for PCa based on patients, and they also reported that the infiltration of T cells and macrophages is increased in the poor outcome group, which is also consistent with our work that M2 macrophages are linked with unfavorable prognosis, while the combination of immunocytes and clinical features could distinguish the different ends of recurrence^25^. Bhargava et al.^26^ illustrated an African-American specifically automated stromal classifier, which has the potential to substantially improve the accuracy of prognosis and risk stratification. Yang et al.^27^ established a 28-hypoxia-related-gene prognostic classifier for localized PCa, which could predict biochemical recurrence and metastasis events. A Gleason score of 4 + 3 is resulted in almost 3-fold metastasis risk at diagnosis compared with a Gleason score of 3 + 4, although the overall incidence is low^28,29^. In our study, we investigated the value of this classifier in distinguishing patients with Gleason score 3 + 4 and 4 + 3 subgroups in the MSKCC, TCGA-PRAD, GSE70768, and GSE70769 datasets. We observed that patients with Gleason score 4 + 3 had a higher-risk score than patients with Gleason score 3 + 4. In addition, the risk score distinguished the 3 + 4/4 + 3 subgroups with good accuracy, a result consistent with Fisher’s extract test. Another limitation of these studies was that their findings were not validated in two more independent cohorts, and the potential mechanisms of how these markers influence tumor progression were not predicted or investigated. Herein, we established an eleven-consensus-gene classifier and validated its usage in five independent cohorts. We also predicted the potential mechanisms through bioinformatic methods. Thus, our findings are stable and convincing.

The advantages of the current study are summarized and presented as follows. First, we identified 39-consensus prognostic genes from five independent cohorts, and with the help of LASSO Cox regression analysis, we chose the eleven most suitable candidates to establish the RFS prediction classifier. The classifier showed satisfying molecular prognostic subtyping accuracy determined by the log-rank, K–M, and ROC analyses in all five cohorts. Second, we further confirmed that the eleven-consensus-gene classifier serves as an independent RFS predictor through multiple platforms and provides a novel method for the prognosis predition. Third, the eleven-consensus-gene classifier is not dependent on the Gleason score as compared with ISUP grading group and EAU risk group, therefore the prediction accuracy will not be impacted by the work experience of pathologist. Forth, we predicted the potential mechanisms of how these critical candidates influence the progression of PCa, which would benefit the development of targeted drugs. However, the lack of survival analysis in our samples and the lack of systematic functional studies to show the function and mechanisms of these consensus genes are the major limitations of the current study.

Our study successfully classified PCa patients with different prognostic outcomes under a consensus ensemble framework using a large clinical cohort of 1046 cases, ending up with an eleven-consensus-gene classifier. The classifier shows comparable prognostic value with ISUP Gleason group and EAU risk group, and also presented a preferable prognostic value after combined with other major clinical features. Comparing with whole-transcriptome profile, target gene profiling panel of small number of genes is wildly applied in the clinical with the high cost-performance ratio. Therefore, the eleven-consensus-gene classifier is promising to be applicable in clinical setting to propel the prognosis prediction for PCa patients.

## Methods

### Data preparation and processing

We searched the Gene Expression Omnibus (GEO) to enroll eligible datasets that met the following criteria: (1) PCa cases with available expression data, and (2) available clinicopathological features, particularly RFS status and time. Then, the gene-expression profiles were generated from four eligible GEO datasets [GSE116918^30^, GSE70769^31^, GSE70768^31^, and GSE21032/Memorial Sloan Kettering Cancer Center (MSKCC)^32^], as well as the gene-expression profile from The Cancer Genome Atlas Prostate Adenocarcinoma (TCGA-PRAD, https://www.cancer.gov/tcga). For gene-expression profile of TCGA-PRAD, the number of fragments per kilobase million (FPKM) was computed and converted into transcripts per kilobase million (TPM) and further log 2 transformed, which showed more similarity to the numbers obtained from microarray analysis and improved comparability between samples, the ensemble IDs were mapped to gene symbols along with the GENCODE 27 file (https://www.gencodegenes.org/human/release_27.html). For the gene symbol with more than one probe IDs, the mean value was calculated as its expression value. We also removed the potential cross-dataset batch effect via the “sva” package along with the empirical Bayes framework^33^. The matched clinicopathological data were also downloaded along with the expression profiles. Patients who lacked pathological T stage data were excluded. We also identified the ISUP groups and EAU risk groups by Gleason score and PSA value^34,35^.

### Univariate Cox regression analysis and consensus-gene selection

We conducted the univariate Cox regression analysis to identify the consensus RFS-related genes from five datasets. Subsequently, we intersected these RFS-related candidates that appeared in all five datasets, with a cutoff *P-*value of less than 0.05. The following analyses were performed based on these selected genes.

### Classifier establishment and validation

According to the results provided by univariate Cox regression analysis, we employed least absolute shrinkage and selection operator (LASSO) Cox regression to select stable prognostic candidates. LASSO is a regression method that uses both regularization and variable selection to elevate the prediction accuracy and interpretability of the results. We calculated the recurrence rate of each cohort, 25.71% for MSKCC, 19.06% for TCGA-PRAD, 17.43% for GSE70768, 48.24% for GSE70769, and 22.87% for GSE116918. We reviewed the published literatures, and acquired that the biochemical recurrence (BCR) rate of localized PCa after radical prostatectomy is about 20–40%^36–38^; therefore, we chose the MSKCC cohort as the training for LASSO analysis. The classifier was established referring to the expression and coefficient of each candidate based on the MSKCC cohort. We computed the risk score for each patient with the following formula:

$${{{{{\mathrm{{risk}}}}}}}\,{{{\mathrm{score}}}}=\mathop{\sum }\limits_{i=1}^{n}[{{{{{\rm{coef}}}}}}\left({{{{{\rm{mRNA}}}}}}i\right)* {{{{{\rm{Expression}}}}}}\left({{{{{\rm{mRNA}}}}}}i\right)]$$

The median value of the risk score was set as the cutoff value in each cohort, and patients with risk scores lower than the median value were assigned to the low-risk subgroup, while others belonged to the high-risk subgroup. The risk score of patients in the other four external validation cohorts, TCGA-PRAD, GSE70768, GSE70769, and GSE116918, was also calculated by this risk formula, and then these patients were dichotomized into two different risk subgroups by the median risk score in each cohort.

### Survival and receiver operating characteristic (ROC) analyses

Survival analyses were executed using the “survminer” package (https://github.com/kassambara/survminer), with BCR as the endpoint. Furthermore, the area under the ROC curve (AUC) was employed to assess the predictive value of the formula. The comparison between two ROC curves was also conducted by the “pROC” package. Furthermore, subgroup analyses were executed to test the accuracy of the classifier in different clinicopathological subgroups, such as different Gleason score (≤7 vs. >7), pathological tumor stage (T1 + T2 vs. T3 + T4), and age (≤60 vs. >60).

### Immunohistochemistry (IHC) validation

To validate the association between SRD5A2 and ITGA11 and the clinicopathological features, we used the IHC assay to detect the protein expression of the above two genes in a prostate cancer tissue array (Outdo Biotech Co., Ltd., Shanghai, China), which contains tumor tissue from 42 patients. The antibodies of SRD5A2 (Cat. #: DF8416, Affinity Biosciences LTD., Ohio, USA) and ITGA11 (Cat. #: bs-13771R, Bioss Antibodies LTD., Massachusetts, USA) were applied for IHC staining at a dilution of 1:250. We recorded the staining intensity as follows: 0, negative; 1, weak positive; 2, moderate positive; and 3, strong positive. In addition, the staining area was indicated as follows: 0, 0%; 1, 1–25%; 2, 26–50%; 3, 51–75%; and 4, >76%. The intensity score multiplied by the staining area was defined as the ultimate score (≥3, positive staining; <3, negative staining)^39^.

### Cell culture and knockdown of SRD5A2 and ITGA11

We cultured the C4-2 and PC-3 cell lines with RPMI 1640 medium, which also contained 10% fetal bovine serum and 1% penicillin and streptomycin, which contained 100 U/ml penicillin and 100 mcg/ml streptomycin. Cells were cultured at 37 °C and 5% CO_2_. PC-3 cell line were kindly provided by Procell Life Science & Technology Co., Ltd (Wuhan, China) and certified by STR profiling cell line authentication (Supplementary Table 2). C4-2 cell line was obtained from Sunncell Bioscience Inc. (Wuhan, China), and certified by STR profiling cell line authentication (Supplementary Table 3). We routinely confirmed that these cell lines were negative for mycoplasma contamination using an e-Myco mycoplasma PCR detection kit (25235; iNtRON Biotechnology, Kirkland, WA, USA). We obtained 1 × 10^8^ TU/ml *shSRD5A2* and *shITGA11* lentiviruses from Shanghai Novobio Co., Ltd. (Shanghai, China). To obtain the lentivirus, shITGA11-1#, shITGA11-2#, shSRD5A2-1#, and shSRD5A2-2# were inserted into the PDS126_pL-U6-shRNA-GFP vector. The knockdown sequences were as follows: *shITGA11-1*#-F: GCTCTTACTTTGGGAGTGAAA, *shITGA11-1*#-R: TTTCACTCCCAAAGTAAGAGC; *shITGA11-2*#-F: GCCATCCAAGATCAACATCTT, *shITGA11-2*#-R: AAGATGTTGATCTTGGATGGC; *shSRD5A2-1*#-F: GTGGTGTCTGCTTAGAGTTTA, *shSRD5A2-1*#-R: TAAACTCTAAGCAGACACCAC; *shSRD5A2-2*#-F: CTCAATCGAGGGAGGCCTTAT, *shSRD5A2-2*#-R: ATAAGGCCTCCCTCGATTGAG. The knockdown cell lines of ITGA11 and SRD5A2 in PC-3 and C4-2 cells were obtained according to the manufacturer’s instructions, 5 µl knockdown lentivirus or control lentivirus was added to each well of a six-well plate, and the cells were treated with ampicillin (50 µg/mL) for 1 month to establish stable ITGA11- or SRD5A2-knockdown cell lines.

### Assay of cell proliferation, migration, and invasion

To evaluate the impact of SRD5A2 and ITGA11 on prostate cancer cells, we employed MTT and colony formation assays to assess the alteration of cell proliferation, while Transwell-based invasion and migration assays were used to evaluate cell migration and invasion.

For the MTT assay, 5000 cells were seeded per well of 24-well plates, and the results were collected by adding 50 μL of prepped 5 mg/mL MTT reagent to 450 μL of refreshed medium (with a concentration of 0.5 mg/mL) and incubating at 37 °C for 1.5 h. We collected the plates on the 0, 2nd, 4th, and 6th days to block cell viability and stored them at −20 °C. On the 6th day, we added DMSO solution to all plates to dissolve the formazan crystals and then read the optical density value at 570 nm to display the cell viability. For colony formation, 800 cells were seeded per well and grown for 12 days. The cells in plates were fixed with 4% paraformaldehyde for 20 min, and 0.05% crystal violet subsequently used to stain these fixed cells for another 20 min.

For the migration assay, Transwell Permeable Supports (Corning Inc., Maine, USA) were used. A total of 1 × 10^5^ cells with FBS-free medium were put into the upper chamber of transwell plate, and 500 μL fresh medium with 10% FBS was filled into the lower chamber. The cells that migrated to the bottom of the membranes were fixed with methanol and further stained with 0.01% crystal violet. The steps of the invasion assay were similar to those of the migration assay, which also used permeable supports but with extra Matrigel (Biocoat, Corning, New York, USA) diluted and coated in the upper chambers and incubated for 36 h. The cell numbers were calculated by counting three random fields.

### Functional prediction

Increasing evidence indicates that highly coexpressed genes potentially have similar biological functions^40–42^, and we identified the coexpressed genes of *ITGA11* in the five cohorts (correlation >0.7) by Pearson correlation analysis. After overlapping these coexpressed genes, we performed Kyoto Encyclopedia of Genes and Genomes (KEGG) pathway enrichment analysis to subclassify their functions based on the “clusterProfiler” R package^43^. In addition, we also used Cytoscape (v3.5.1, San Diego, La Jolla, California, USA) to visualize the functional network. Two external gene sets, HALLMARK PI3K/AKT SIGNALING and REACTOME PI3K/AKT ACTIVATION, were employed to assess the association between ITGA11 expression and the activation of the PI3K signaling pathway. Single-sample gene set enrichment analysis (ssGSEA)^44,45^, implemented in the GSVA R package, was applied to calculate the normalized enrichment score (NES) of the above 2 gene sets. For a gene matrix, the enrichment score (ES) reflects the degree to which a gene set is overrepresented at the top or bottom of a ranked list of genes, and NES means the corrects for differences in ES between gene sets due to differences in gene set sizes, NES was calculated with below formula:

$${{{{{{\mathrm{NES}}}}}}}=\frac{{{{{{{\mathrm{Actual}}}}}}}\,{{{{{{\mathrm{ES}}}}}}}}{{{{{{{\mathrm{Mean}}}}}}}\,({{{{{{\mathrm{ESs}}}}}}}\,{{{{{{\mathrm{against}}}}}}}\,{{{{{{\mathrm{all}}}}}}}\,{{{{{{\mathrm{permutations}}}}}}}\,{{{{{{\mathrm{of}}}}}}}\,{{{{{{\mathrm{the}}}}}}}\,{{{{{{\mathrm{dataset}}}}}}})}$$

### Western blot validation

For the C4-2 and PC-3 cell lines with or without knockdown of SRD5A2 and ITGA11, we collected the cells at log phase and lysed cells with RIPA lysis buffer (Cat. #P0013B, Beyotime, Shanghai, China) added protease inhibitor and phosphatase inhibitor (Cat. #P1405, Beyotime, Shanghai, China). Proteins (40–50 μg) were separated on 12.5% SDS/PAGE gels and then transferred onto nitrocellulose blotting membranes (GE Healthcare Life Science, Germany). Membranes were blocked with 5% bovine serum albumin (Sigma–Aldrich, St. Louis, MO, USA) for 1 h at room temperature and then incubated with appropriate dilutions of specific primary antibodies against SRD5A2 (Cat. #: DF8416, Affinity Biosciences LTD., Ohio, USA), ITGA11 (Cat. #: bs-13771R, Bioss Antibodies LTD., Massachusetts, USA), AKT1/2/3 (Cat. #: AF6216, Affinity Biosciences LTD., Ohio, USA), p-AKT1/2/3 (Ser^473^) (Cat. #: AF0016, Affinity Biosciences LTD., Ohio, USA), GAPDH (Cat. #: 1049-1-AP, Proteintech Group, Illinois, USA) overnight at 4 °C. The next day, after incubation with HRP-conjugated secondary antibodies for one hour, the membranes were visualized using an ECL system (Pierce; Thermo Fisher Scientific, Inc., USA).

### Collection of proposed prognostic signatures

To assess the prognostic value of the eleven-consensus-gene classifier with other tools, we collected the formula of proposed signatures. Liu et al.^46^ reported a 13-stem call-associated signature, with the formula: Risk score = (0.245 × expression level of BMP8B) + (0.630 × expression level of BOD1) + (0.446 × expression level of CTNNBIP1) + (−0.594 × expression level of FZD5) + (0.207 × expression level of GREM1) + (−0.265 × expression level of LATS2) + (0.349 × expression level of NAMPT) + (− 0.263 × expression level of PRKACB) + (0.342 × expression level of RBPJL) + (− 0.076 × expression level of SEL1L) + (0.825 × expression level ofSTK36) + (0.05 × expression level of TCF15) + (0.287 × expression level of WNT4). Zhang et al.^47^ reported a PCSS score with 13 genes as well, with the formula: PCSS = − 0.39233*ASF1B-0.21563*AURKB-0.02372*CCNA2 + 0.12167*CDC20–0.38666*CDKN3 + 0.73003*CHTF18 + 0.05862*EZH2-0.26846*FOXM1 + 1.40193*KIF4A + 0.10177*MYBL2 + 0.7149*PLK1 + 0.30036*PTTG1–1.10124*TRIP13. The CCP score^48^ was calculated by 31 CCP genes (FOXM1, ASPM, TK1, PRC1, CDC20, BUB1B, PBK, DTL, CDKN3, RRM2, ASF1B, CEP55, CDC2, DLGAP5, C18orf24, RAD51, KIF11, BIRC5, RAD54L, CENPM, KIAA0101, KIF20A, PTTG1, CDCA8, NUSAP1, PLK1, CDCA3, ORC6L, CENPF, TOP2A, MCM10) and 15 housekeeping genes (RPL38, UBA52, PSMC1, RPL4, RPL37, RPS29, SLC25A3, CLTC, TXNL1, PSMA1, RPL8, MMADHC, RPL13A, PPP2CA, MRFAP1). The CCP score = average expression of 31 CCP genes/average expression of 15 housekeeping genes.

### Statistics and reproducibility

All comparisons of continuous data among two subtypes were performed by Student’s *t*-test and Mann–Whitney U test for normally and nonnormally distributed data. Correlations between staining intensity subgroups and clinicopathological subgroups were evaluated by Fisher’s exact test. Spearman’s correlation analysis was utilized to explore the correlation between continuous variables. One-way analysis of variance (ANOVA) was used for the comparison of more than two groups. For all statistical analyses, a two-tailed *P*-value less than 0.05 was considered statistically significant. All experiments were taken from distinct samples and the number of biological replicates (n) is indicated in figure legends. Flow chart for the steps applied in the current study demonstrated in Fig. 7b.

### Reporting summary

Further information on research design is available in the Nature Research Reporting Summary linked to this article.

## Supplementary information

Supplementary Information

Description of Additional Supplementary Files

Supplementary Data 1

Reporting Summary

## Data Availability

All data used in this work can be acquired from the Gene-Expression Omnibus (GEO; https://www.ncbi.nlm.nih.gov/geo/) and the GDC portal (https://portal.gdc.cancer.gov/). Uncropped and unedited blot/gel images are listed in Supplementary Fig. 6.

## Code availability

All the original R codes are available from the corresponding author upon reasonable request.
