# Supplementary material for: Risk subtyping and prognostic assessment of prostate cancer based on consensus genes
Source: Commun Biol. 2022 Mar 15;5:233. doi: 10.1038/s42003-022-03164-8 (PMC8924191; doi:10.1038/s42003-022-03164-8)
Supplement: Supplementary file 1 — Supplementary Information [file 42003_2022_3164_MOESM1_ESM.pdf]

Supplementary Information

Risk subtyping and prognostic assessment of prostate cancer based on consensus genes

Jialin Meng[1†], Yu Guan[1†], Bijun Wang[1], Lei Chen[1], Junyi Chen[1], Meng Zhang[1,2*], Chaozhao Liang[1*]

[1]Department of Urology, The First Affiliated Hospital of Anhui Medical University, 218th Jixi Road, Hefei 230022, Anhui, People's Republic of China

[2]Institute of Urology, Anhui Medical University, 218th Jixi Road, Hefei 230022, Anhui, People's Republic of China

[3]Anhui Province Key Laboratory of Genitourinary Diseases, Anhui Medical University, 218th Jixi Road, Hefei 230022, Anhui, People's Republic of China

[4]Urology Institute of Shenzhen University, The Third Affiliated Hospital of Shenzhen University, Shenzhen University, Shenzhen 518000, Guangdong, People's Republic of China.

[†]These authors contributed equally.

Running title: Molecular prognostic assessment of prostate cancer

***Corresponding author:** Meng Zhang (zhangmeng1930@126.com) & Chaozhao Liang (liang_chaozhao@ahmu.edu.cn).

Tel.: +86 19955195611, and **Fax.:** +86 19955195611

Address: Jixi Road 218, Shushan District, Hefei City 230022, Anhui Province, People's Republic of China.

Supplementary Information includes:
Supplementary Figures 1 – 8
Supplementary Tables 1 – 4

Supplementary Figures

Supplementary Figure 1

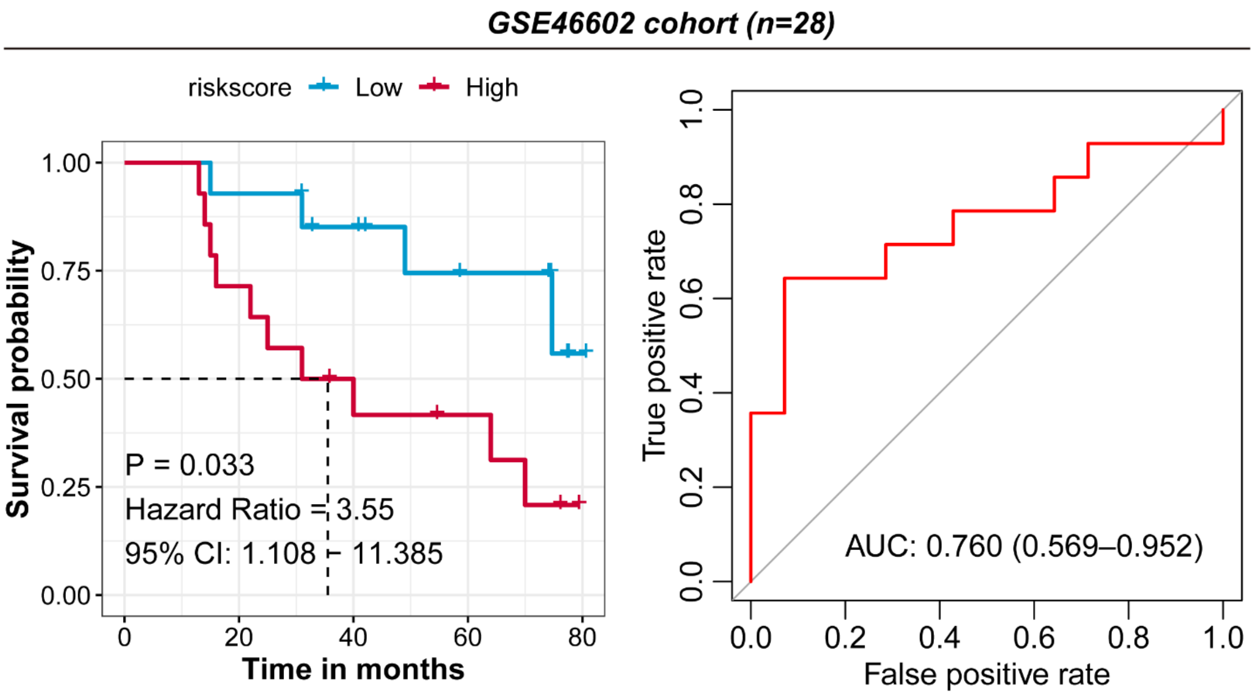

Supplementary Figure 1.
Prognostic value of eleven-consensus-gene-based classifier validated in external GSE46602 cohort.

Supplementary Figure 2 (page 1)

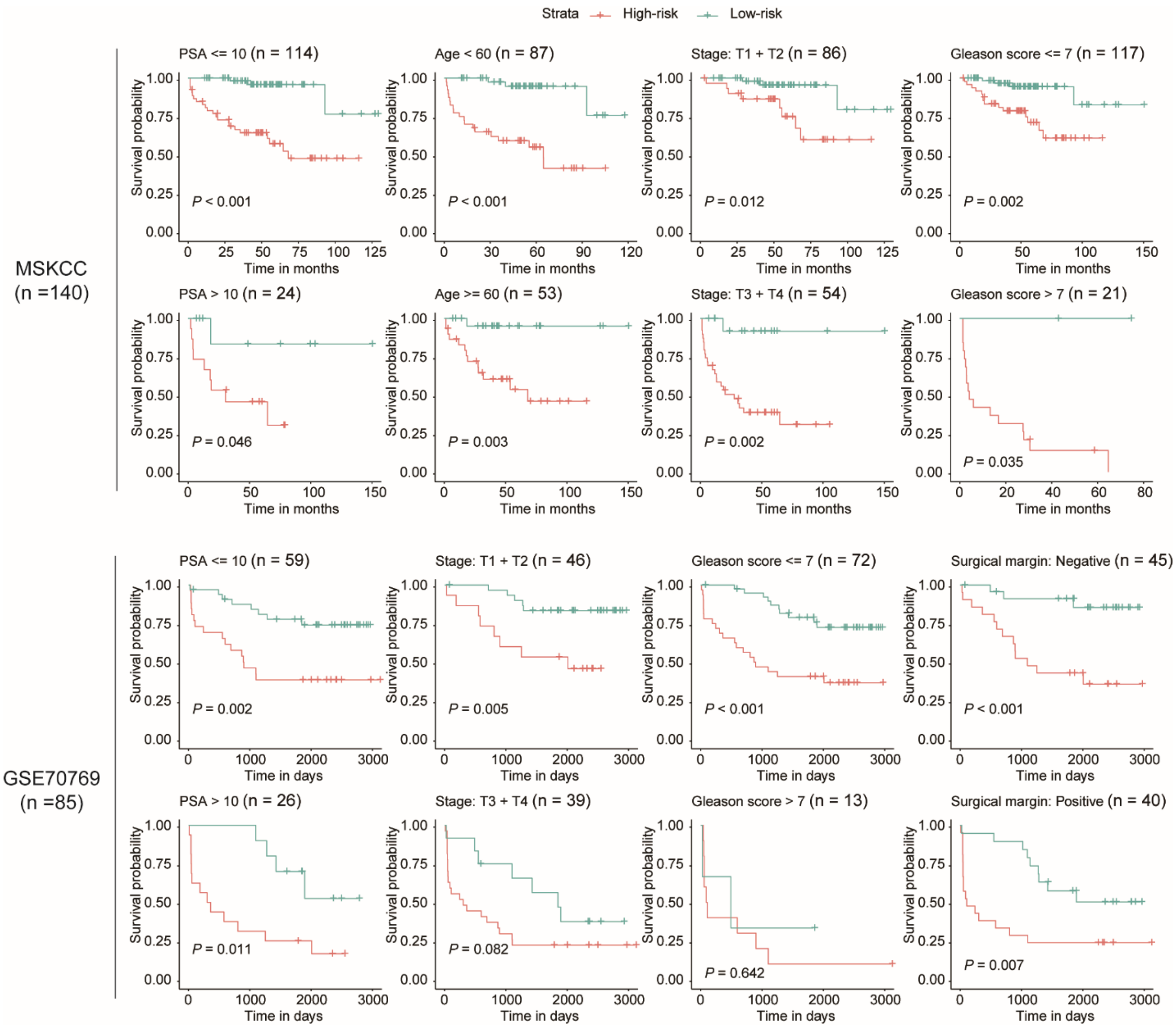

Supplementary Figure 2 (page 2)

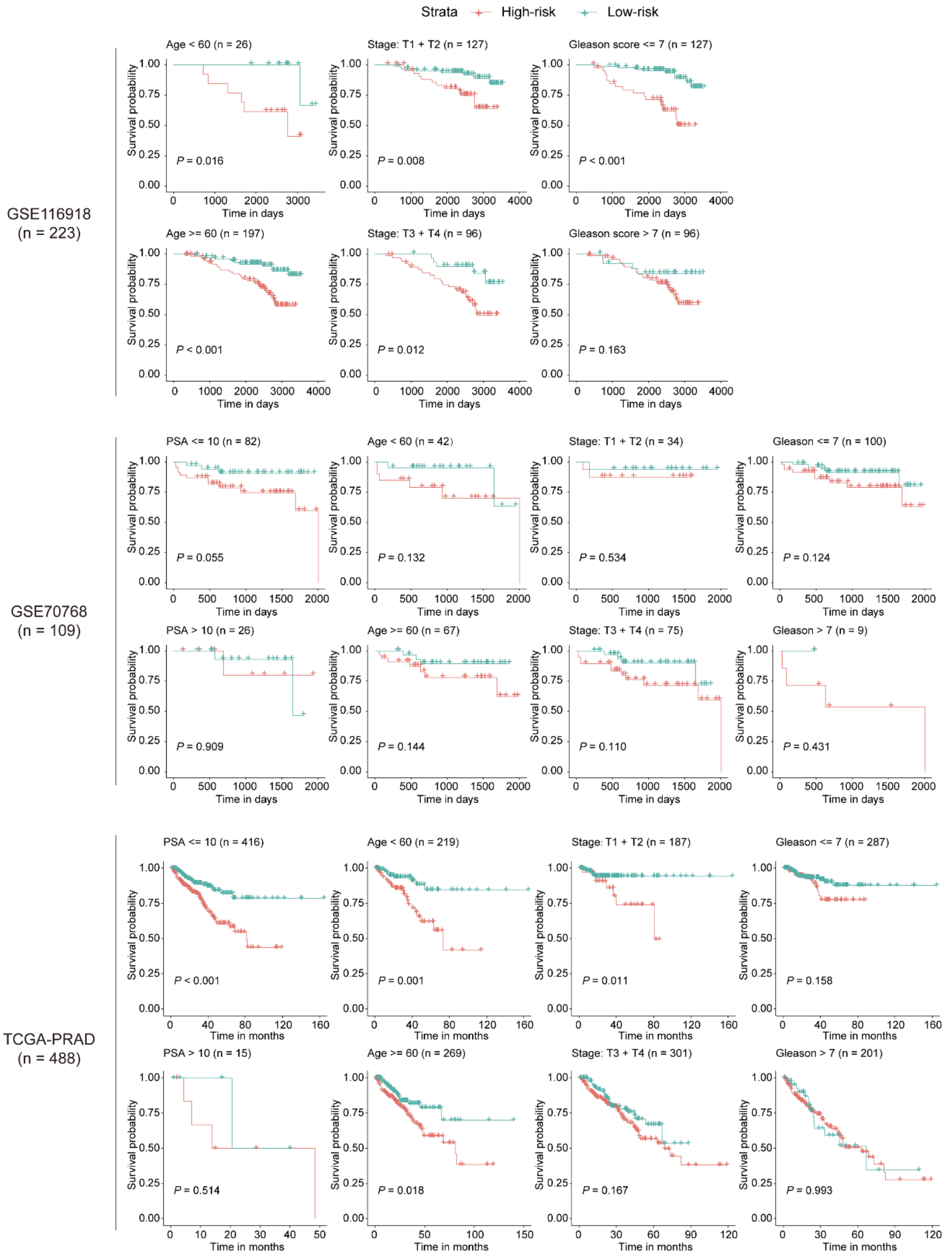

Supplementary Figure 2.
Kaplan-Meier analyses showed the usage of the eleven-consensus-gene-based classifier in different clinicopathological subgroups in five cohorts. Survival probability reflected the recurrence-free survival, P value obtained from the log-rank test.

Supplementary Figure 3

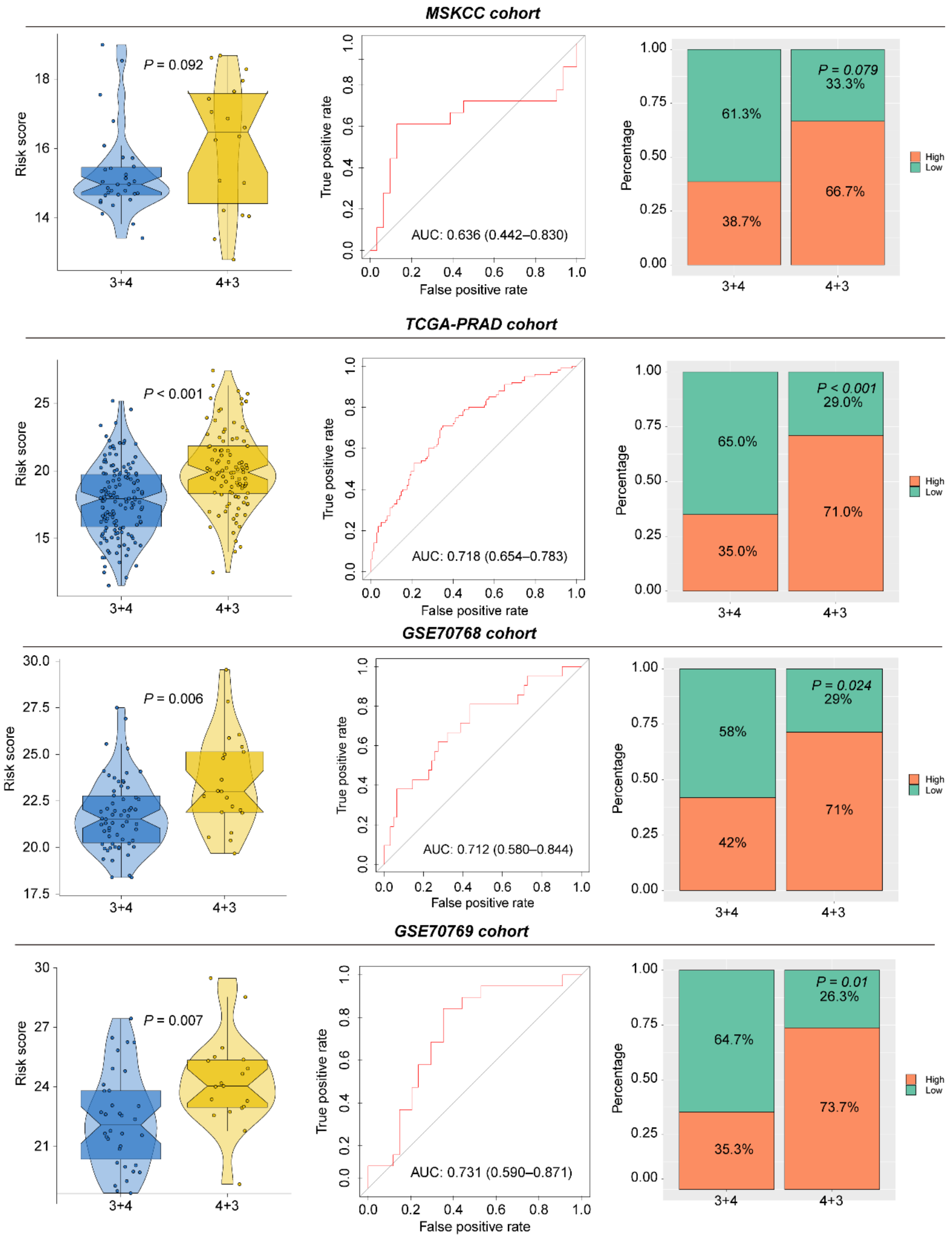

Supplementary Figure 3.
Classifier signature could distinguish patients with 3+4/4+3 Gleason score.
Student's T-test compared the patients' risk score in 3+4/4+3 Gleason score subgroups; ROC curve and AUC value assessed the predicted accuracy; and Fisher's extract test evaluated the distribution of high-risk or low-risk patients in 3+4/4+3 Gleason score subgroups.

Supplementary Figure 4

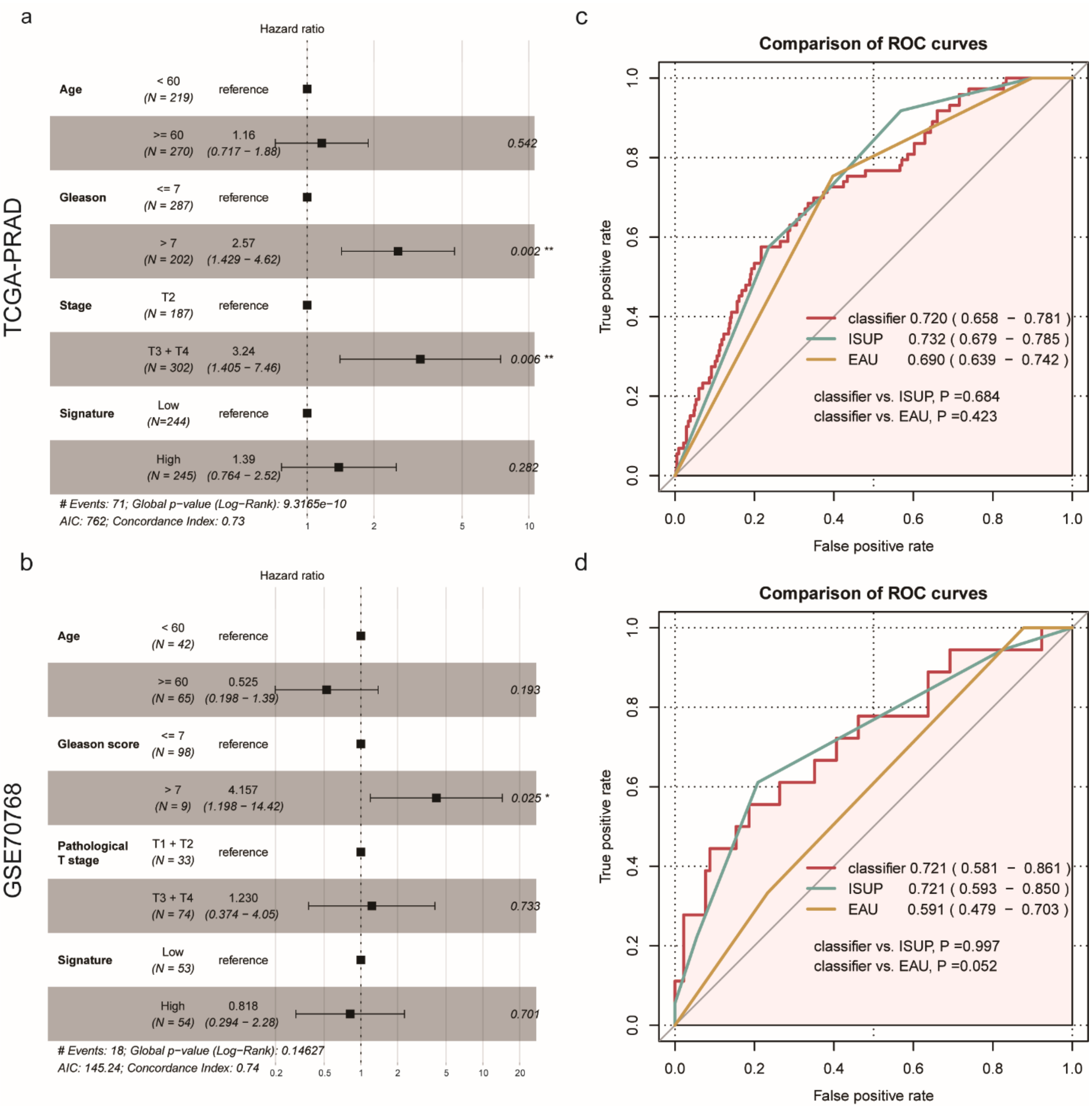

Supplementary Figure 4.
Multivariate regression and combined receiver operating characteristic curve analyses. Forest plot showing the independent prognostic value of the classifier in the TCGA-PRAD (a), and GSE70768 (b) cohorts; Comparison of the prognostic value of clinicopathological features, classifier and the synthesized model by receive operating characteristic curve in the TCGA-PRAD (c), and GSE70768 (d) cohorts.

Supplementary Figure 5

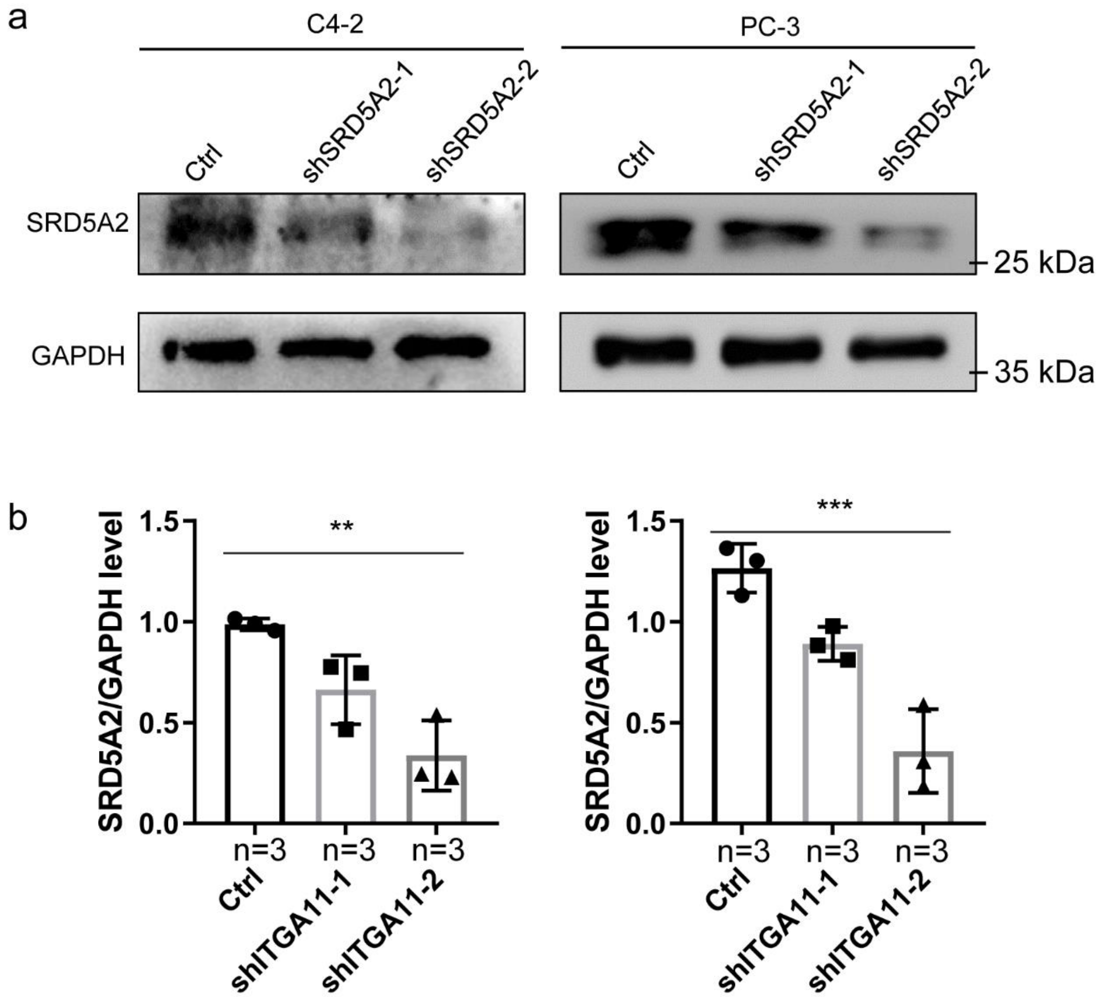

Supplementary Figure 5.
Knockdown efficiencies of SRD5A2 in prostate cancer C4-2 and PC-3 cell lines.
(a) Western blotting showing the knockdown of SRD5A2 in C4-2 and PC-3; (b) quantification of the western blotting results showing in (a).

Supplementary Figure 6 (page 1)

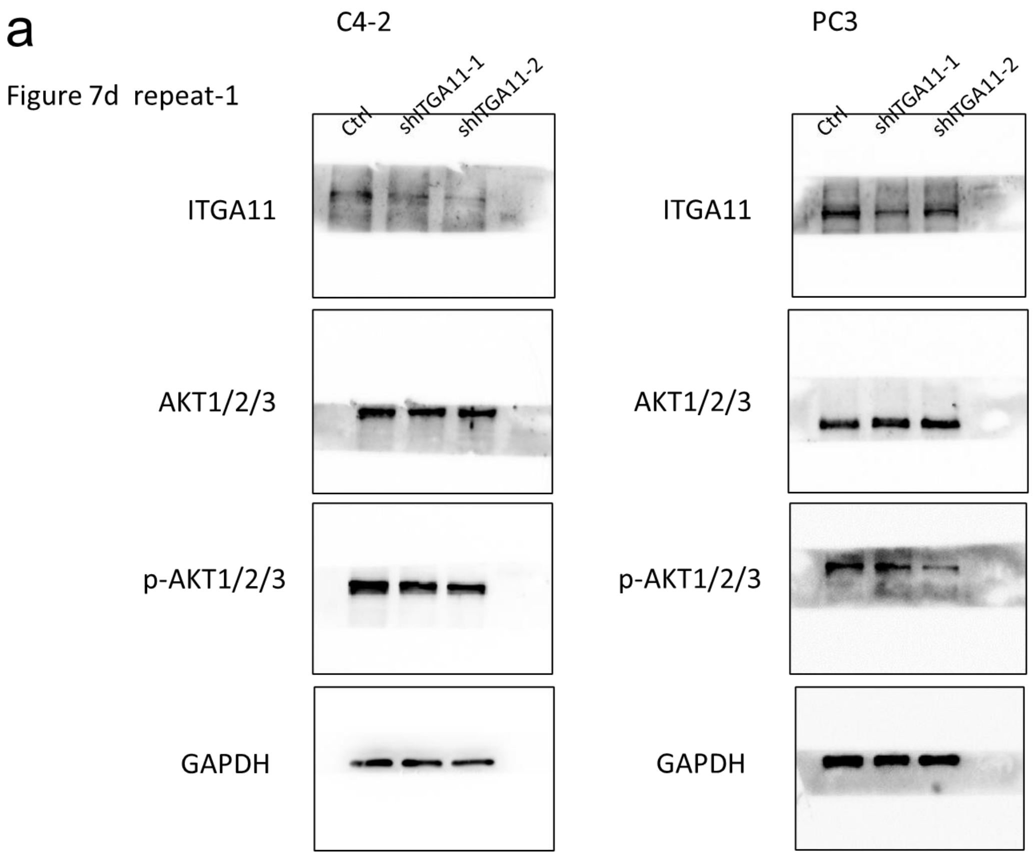

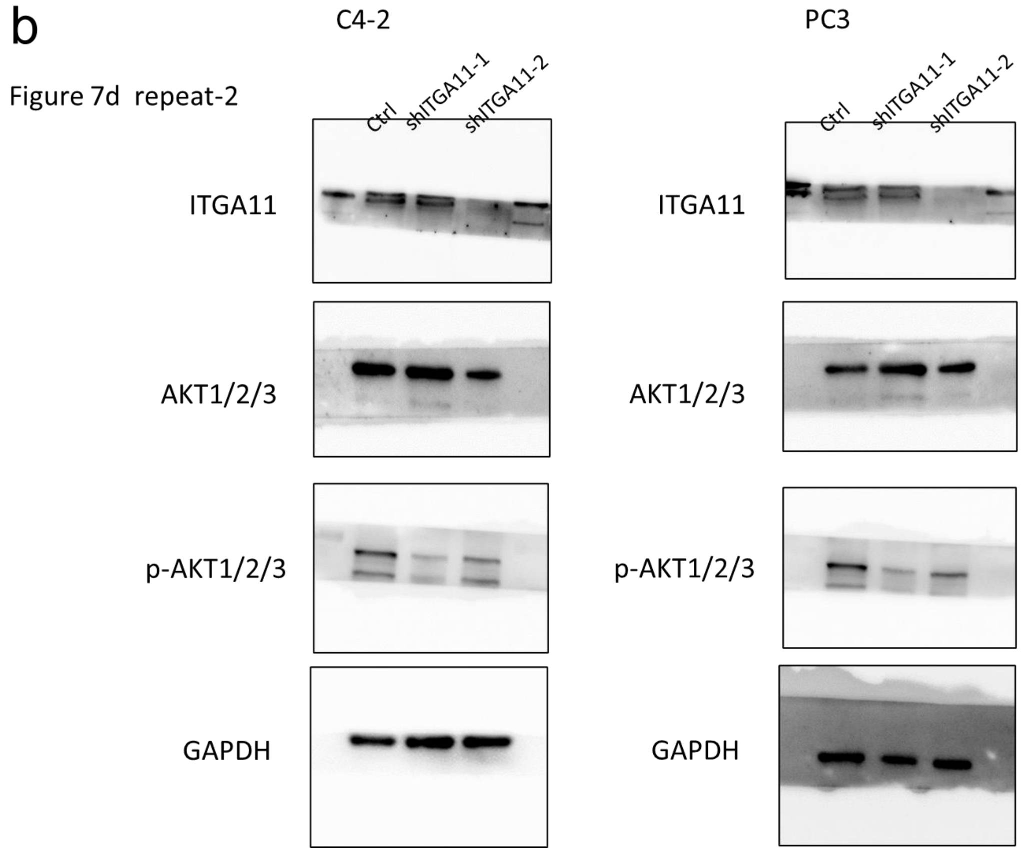

Supplementary Figure 6 (page 2)

c

Figure 7d repeat-3

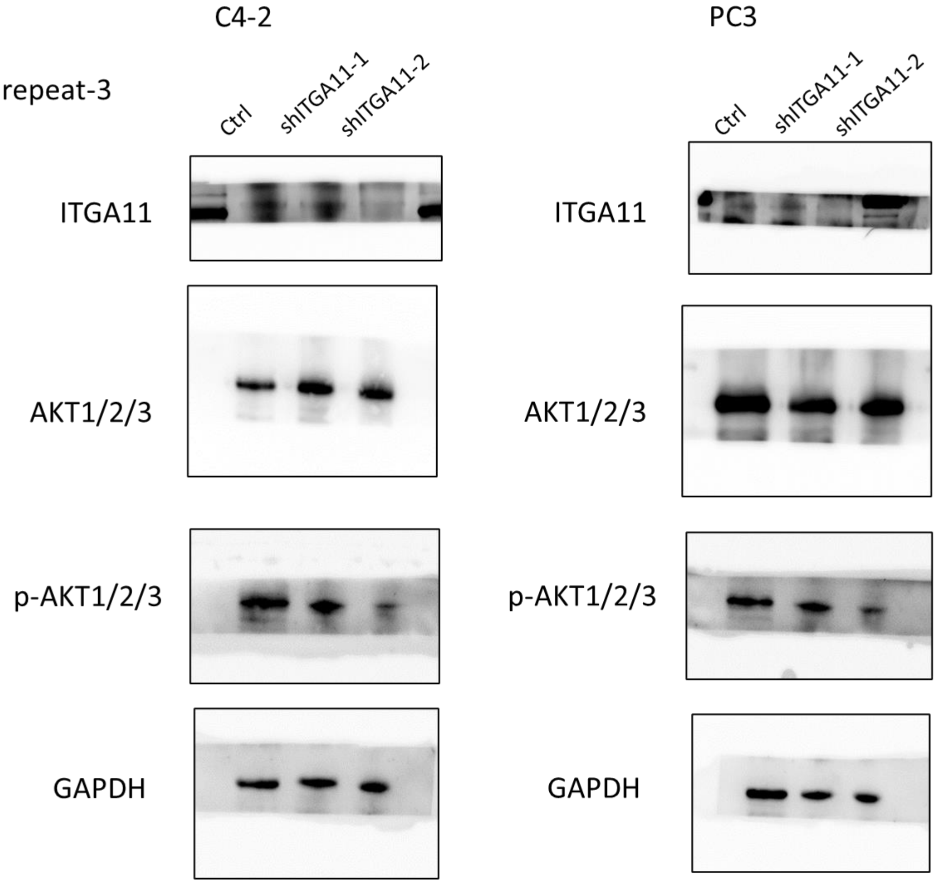

d

Figure S6 repeat-1

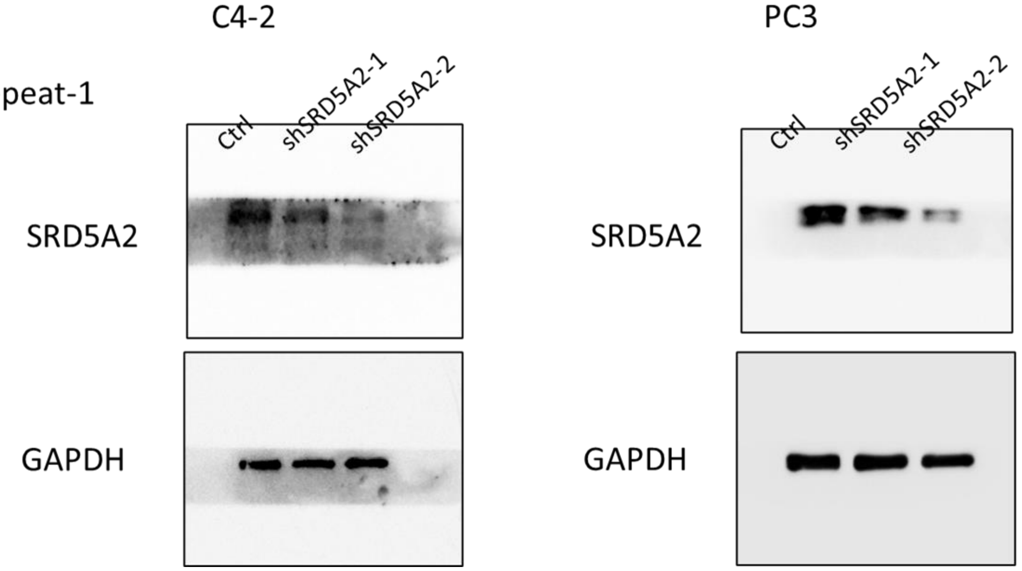

e

Figure S6 repeat-2

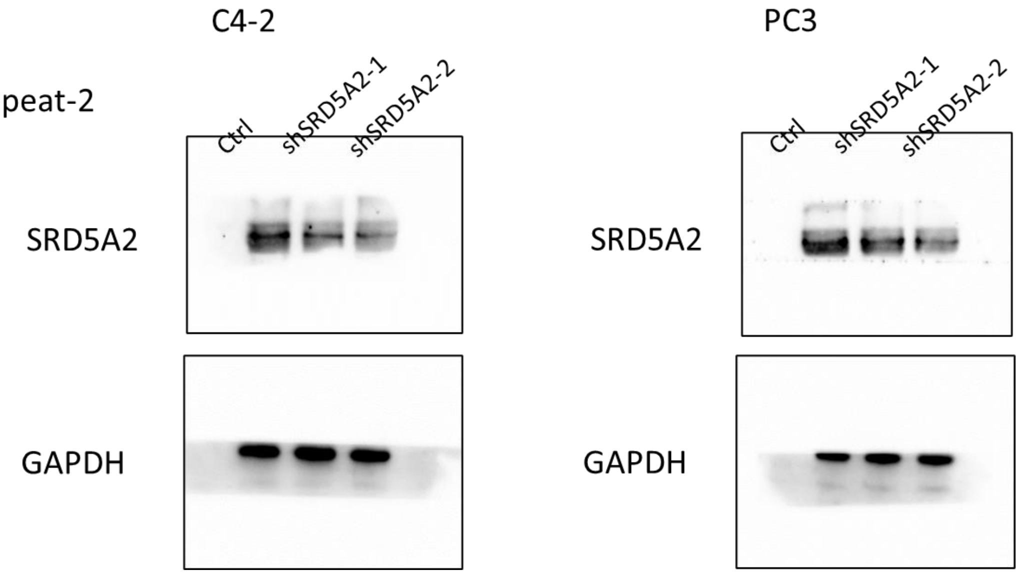

f

Figure S6 repeat-3

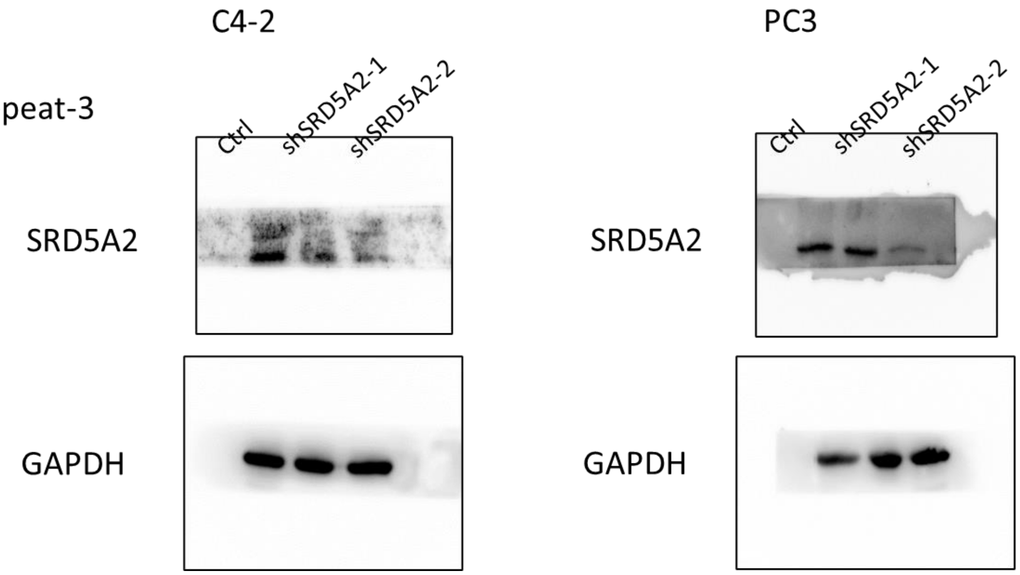

Supplementary Figure 6.
Original lanes of western blotting showing in Figure 7D and Figure S5.
(a) immunoblots for Figure 7d repeat 1; (b) immunoblots for Figure 7d repeat 2; (c) immunoblots for Figure 7d repeat 3; (d) immunoblots for Supplementary Figure 6a repeat 1; (e) immunoblots for Supplementary Figure 6a repeat 2; (f) immunoblots for Supplementary Figure 6a repeat 3.

Supplementary Tables

Supplementary Table 1. The univariate Cox regression analyses results derived from five independent datasets.

Gene ID	Type	TCGA		MSKCC		GSE116918		GSE70769		GSE70768	
		Hazard ratio	***P*-value**	***Hazard ratio***	***P*-value**	***Hazard ratio***	***P*-value**	***Hazard ratio***	***P*-value**	***Hazard ratio***	***P*-value**
ANLN	Risky	1.818	0.000585	3.473	1.56E-08	1.87	0.007743	34.7	0.000495	19.836	0.049352
ANO4	Protective	0.595	0.000646	0.395	0.023303	0.614	0.033051	0.012	0.000183	0.063	0.00443
ASPM	Risky	2.315	0.002734	4.477	1.25E-06	3.432	0.000265	9.516	0.000719	3.043	0.034281
CDC20	Risky	2.15	0.000737	8.545	9.60E-06	2.419	0.011485	5.29	4.87E-05	2.596	5.01E-05
CDKN3	Risky	2.12	0.003794	12.901	2.57E-08	1.82	0.031737	9.52	0.003273	2.462	0.011557
CENPF	Risky	1.853	0.001031	3.455	1.91E-08	2.273	0.005618	17.347	0.000904	7.094	7.34E-05
CHRDL1	Protective	0.752	0.022902	0.444	1.71E-06	0.827	0.018452	0.007	0.010462	0.016	0.001669
COL1A1	Risky	1.578	0.002453	2.747	9.00E-06	1.993	8.85E-05	1.757	0.000497	1.781	0.033756
CRIP2	Risky	1.886	0.017128	2.222	0.000277	2.637	0.009705	1.899	0.000698	1.613	0.004139
DPP4	Protective	0.707	0.004453	0.641	4.55E-06	0.798	0.029369	0.238	0.000685	0.558	0.005094
EPHX2	Protective	0.498	8.21E-05	0.405	2.57E-05	0.575	0.017721	0.264	0.001298	0.309	0.001175
EXO1	Risky	2.499	3.55E-05	4.085	3.48E-06	2.336	0.000201	1151.588	0.009279	6.568	0.024098
HEATR2	Risky	2.081	0.049992	8.715	0.00251	1.726	0.008369	27.561	0.009506	10.47	0.043913
ITGA11	Risky	1.61	0.002133	8.705	0.000126	2.363	0.002034	8.596	0.000862	4.649	0.002114
ITM2C	Protective	0.649	0.024814	0.305	0.003513	0.508	0.026088	0.263	0.00822	0.506	0.012089
KIF13B	Protective	0.64	0.041185	0.206	1.69E-06	0.833	0.022443	0.33	0.00417	0.33	0.04153
KIF14	Risky	2.783	0.000288	5.464	4.02E-05	1.512	0.027044	30.793	0.004841	9.389	0.015271
KIF20A	Risky	1.954	0.00209	4.794	5.28E-07	1.935	0.010647	19.623	0.000249	3.587	0.005952
LRFN4	Risky	2.618	0.038322	2.839	0.038288	1.987	0.031225	4.836	0.009195	3.972	0.040673
LTBP2	Risky	1.455	0.022957	5.942	0.015248	2.148	0.00028	2.743	0.00212	2.185	0.016731
MMP11	Risky	1.67	3.37E-05	6.305	0.000268	1.959	8.76E-05	8.235	0.000493	2.35	2.51E-06
MT1F	Protective	0.719	0.000444	0.452	0.001648	0.782	0.006238	0.606	0.043437	0.8	0.031284
MT1H	Protective	0.655	0.005775	0.354	0.000602	0.79	0.034542	0.241	0.004367	0.54	0.00809
MYBPC1	Protective	0.73	0.030094	0.548	4.63E-09	0.769	0.023345	0.376	5.94E-05	0.475	0.020869

NOX4	Risky	1.897	0.022536	2.368	0.002576	2.008	8.19E-05	5.104	0.039666	2.049	0.033975
NUSAP1	Risky	1.955	0.000352	4.468	5.14E-08	3.086	0.000879	12.093	0.000731	2.499	0.003622
OGN	Protective	0.731	0.022552	0.293	7.48E-08	0.82	0.036713	0.165	0.025742	0.435	0.016997
PAGE4	Protective	0.687	0.000461	0.207	0.016862	0.842	0.037535	0.392	1.36E-05	0.624	6.47E-05
PEBP4	Protective	0.814	0.02294	0.457	0.004198	0.435	0.015831	0.353	0.003794	0.585	0.014713
PRR7	Risky	2.194	0.028593	5.879	0.005917	1.408	0.017452	4.246	0.000374	2.235	0.020167
SRD5A2	Protective	0.611	0.000162	0.135	3.15E-09	0.345	3.51E-05	0.13	1.51E-05	0.181	0.001218
SRPX	Protective	0.698	0.02499	0.304	0.039118	0.832	0.002475	0.339	0.032564	0.548	0.006356
STMN1	Risky	2.432	0.013718	5.693	7.66E-08	1.989	0.02124	262.902	0.000124	36.756	0.024222
TMEM132A	Risky	1.821	0.009777	7.3	5.01E-05	2.605	0.040191	3.535	0.000121	2.408	0.025885
TOP2A	Risky	1.614	0.00228	2.021	2.03E-07	2.36	0.01652	3.446	0.000252	2.15	0.000762
TPX2	Risky	2.002	0.001077	2.598	1.90E-08	2.554	0.000105	42.237	6.44E-05	7.062	2.73E-07
UBE2C	Risky	1.888	0.000926	4.03	1.03E-07	3.13	0.001493	16.655	1.29E-06	5.231	0.000771
UBE2J1	Protective	0.656	0.019288	0.513	0.022882	0.364	0.019187	0.283	0.010302	0.479	0.044819
ZNF467	Risky	2.171	0.001858	9.384	0.00011	2.184	0.005714	2.237	0.001723	1.909	0.038767

Note: Risky, means gene expression positively correlated to the RFS prognosis; Protective, means gene expression negatively correlated to the RFS prognosis

Supplementary Table 2. Information of certified PC-3 cell lines by STR.

STR Loci	PC-3 cell sample	Dataset record of PC-3
Amelogenin	X	X
CSF1PO	11	11
D2S1338	18,20	18,20
D3S1358	16	16
D5S818	13	13
D7S820	8,11	8,11
D8S1179	13	13
D13S317	11	11
D16S539	11	11
D18S51	14,15	14,15
D19S433	14	14
D21S11	29,31.2	29,31.2
FGA	24	24
PentaD	9	9
PentaE	10,17	10,17
TH01	6,7	6,7
TPOX	8,9	8,9
vWA	17	17

Supplementary Table 3. Information of certified C4-2 cell lines by STR.

STR Loci	C4-2 cell sample	dataset record of LNCaP*
D5S818	11,12	11,12
D13S317	10,11,12,13	10,12
D7S820	9.1,9.3,10.3	9.1,10.3
D16S539	11,11	11,11
VWA	16,18,19	16,18
THO1	9,9	9,9
AMEL	X,Y	X,Y
TPOX	8,9	8,9
CSFIPO	10,11,12	10,11

*C4-2 cell line is a derivative subline of human prostate cancer LNCaP cell line.
