# Supplementary material for: Risk subtyping and prognostic assessment of prostate cancer based on consensus genes
Source: Commun Biol. 2022 Mar 15;5:233. doi: 10.1038/s42003-022-03164-8 (PMC8924191; doi:10.1038/s42003-022-03164-8)
Supplement: Supplementary file 2 — Description of Additional Supplementary Files [file 42003_2022_3164_MOESM2_ESM.pdf]

Description of Additional Supplementary Files

File name: Supplementary Data 1
Description: Risk subclassification of prostate cancer patients.
