# Supplementary material for: Risk subtyping and prognostic assessment of prostate cancer based on consensus genes
Source: Commun Biol. 2022 Mar 15;5:233. doi: 10.1038/s42003-022-03164-8 (PMC8924191; doi:10.1038/s42003-022-03164-8)
Supplement: Supplementary file 4 — Reporting Summary [file 42003_2022_3164_MOESM4_ESM.pdf]

nature portfolio

Corresponding author(s): Chaozhao Liang

Last updated by author(s): Jan 6, 2022

Reporting Summary

Nature Portfolio wishes to improve the reproducibility of the work that we publish. This form provides structure for consistency and transparency in reporting. For further information on Nature Portfolio policies, see our Editorial Policies and the Editorial Policy Checklist.

Statistics

For all statistical analyses, confirm that the following items are present in the figure legend, table legend, main text, or Methods section.

n/a	Confirmed	
☐	☒	The exact sample size (*n*) for each experimental group/condition, given as a discrete number and unit of measurement
☐	☒	A statement on whether measurements were taken from distinct samples or whether the same sample was measured repeatedly
☐	☒	The statistical test(s) used AND whether they are one- or two-sided<br>*Only common tests should be described solely by name; describe more complex techniques in the Methods section.*
☐	☒	A description of all covariates tested
☐	☒	A description of any assumptions or corrections, such as tests of normality and adjustment for multiple comparisons
☐	☒	A full description of the statistical parameters including central tendency (e.g. means) or other basic estimates (e.g. regression coefficient) AND variation (e.g. standard deviation) or associated estimates of uncertainty (e.g. confidence intervals)
☐	☒	For null hypothesis testing, the test statistic (e.g. *F*, *t*, *r*) with confidence intervals, effect sizes, degrees of freedom and *P* value noted<br>*Give P values as exact values whenever suitable.*
☒	☐	For Bayesian analysis, information on the choice of priors and Markov chain Monte Carlo settings
☒	☐	For hierarchical and complex designs, identification of the appropriate level for tests and full reporting of outcomes
☒	☐	Estimates of effect sizes (e.g. Cohen's *d*, Pearson's *r*), indicating how they were calculated

Our web collection on statistics for biologists contains articles on many of the points above.

Software and code

Policy information about availability of computer code

Data collection	All data used in this work can be acquired from the Gene-Expression Omnibus (GEO; https://www.ncbi.nlm.nih.gov/geo/) and the GDC portal (https://portal.gdc.cancer.gov/).
Data analysis	All comparisons of continuous data among two subtypes were performed by Student's t-test and Mann–Whitney U test for normally and nonnormally distributed data. Correlations between staining intensity subgroups and clinicopathological subgroups were evaluated by Fisher's exact test. Spearman's correlation analysis was utilized to explore the correlation between continuous variables. For all statistical analyses, a two-tailed P value less than 0.05 was considered statistically significant.

For manuscripts utilizing custom algorithms or software that are central to the research but not yet described in published literature, software must be made available to editors and reviewers. We strongly encourage code deposition in a community repository (e.g. GitHub). See the Nature Portfolio guidelines for submitting code & software for further information.

Data

Policy information about availability of data

All manuscripts must include a data availability statement. This statement should provide the following information, where applicable:

- Accession codes, unique identifiers, or web links for publicly available datasets
- A description of any restrictions on data availability
- For clinical datasets or third party data, please ensure that the statement adheres to our policy

All data used in this work can be acquired from the Gene-Expression Omnibus (GEO; https://www.ncbi.nlm.nih.gov/geo/) and the GDC portal (https://portal.gdc.cancer.gov/). All the original R codes are available from the corresponding author on reasonable request.

Field-specific reporting

Please select the one below that is the best fit for your research. If you are not sure, read the appropriate sections before making your selection.

☒ Life sciences ☐ Behavioural & social sciences ☐ Ecological, evolutionary & environmental sciences

For a reference copy of the document with all sections, see nature.com/documents/nr-reporting-summary-flat.pdf

Life sciences study design

All studies must disclose on these points even when the disclosure is negative.

Sample size	In the current study, we enrolled a total of 1,046 PCa patients from the MSKCC (n = 140), TCGA-PRAD (n = 489), GSE116918 (n = 223), GSE70769 (n = 85), GSE70768 (n = 109) datasets. And we used IHC assay on prostate cancer tissue array, which contains tumor tissues from 42 patients.
Data exclusions	Patients who lack the data of the pathology T stage were excluded.
Replication	We provide detailed experimental methods, cell types and antibody catalogue number
Randomization	NA
Blinding	The investigators were blinded to group allocation during data collection or analysis

Reporting for specific materials, systems and methods

We require information from authors about some types of materials, experimental systems and methods used in many studies. Here, indicate whether each material, system or method listed is relevant to your study. If you are not sure if a list item applies to your research, read the appropriate section before selecting a response.

Materials & experimental systems

n/a	Involved in the study	
☐	☒	Antibodies
☐	☒	Eukaryotic cell lines
☒	☐	Palaeontology and archaeology
☒	☐	Animals and other organisms
☒	☐	Human research participants
☒	☐	Clinical data
☒	☐	Dual use research of concern

Methods

n/a	Involved in the study	
☒	☐	ChIP-seq
☒	☐	Flow cytometry
☒	☐	MRI-based neuroimaging

Antibodies

Antibodies used	SRD5A2 (Cat. #: DF8416, Affinity Biosciences LTD., Ohio, USA) and ITGA11 (Cat. #: bs-13771R, Bioss Antibodies LTD., Massachusetts, USA) AKT1/2/3 (Cat. #: AF6216, Affinity Biosciences LTD., Ohio, USA), p-AKT1/2/3 (Ser473) (Cat. #: AF0016, Affinity Biosciences LTD., Ohio, USA), GAPDH (Cat. #: 1049-1-AP, Proteintech Group, Illinois, USA)
Validation	All antibody verification can be found on the company's official website based on the serial numbers

Eukaryotic cell lines

Policy information about cell lines

Cell line source(s)	PC-3: Procell Life Science & Technology Co., Ltd (Wuhan, China); C4-2: ATCC (CRL-3314, Manassas, VA, USA)
Authentication	PC-3 cell line was certified by STR profiling cell line authentication (Supplementary Table 3).
Mycoplasma contamination	We routinely confirmed that these cell lines were negative for mycoplasma contamination using an e-Myco mycoplasma PCR detection kit (25235; iNtRON Biotechnology, Kirkland, WA, USA).
Commonly misidentified lines (See ICLAC register)	*Name any commonly misidentified cell lines used in the study and provide a rationale for their use.*
